# Application of *Rhodococcus jostii* RHA1 glycolate oxidase as an efficient accessory enzyme for lignin conversion by bacterial Dyp peroxidase enzymes†

**DOI:** 10.1039/d3gc00475a

**Published:** 2023-04-11

**Authors:** Awatif Alruwaili, Goran M. M. Rashid, Timothy D. H. Bugg

**Affiliations:** a Department of Chemistry, University of Warwick Coventry CV4 7AL UK T.D.Bugg@warwick.ac.uk

## Abstract

Lignin oxidation by bacterial dye-decolorizing peroxidase enzymes requires hydrogen peroxide as a co-substrate, an unstable and corrosive oxidant. We have identified a glycolate oxidase enzyme from *Rhodococcus jostii* RHA1 that can couple effectively at pH 6.5 with DyP peroxidase enzymes from *Agrobacterium* sp. or *Comamonas testosteroni* to oxidise lignin substrates without addition of hydrogen peroxide. *Rhodococcus jostii* RHA1 glycolate oxidase (RjGlOx) has activity for oxidation of a range of α-ketoaldehyde and α-hydroxyacid substrates, and is also active for oxidation of hydroxymethylfurfural (HMF) to furandicarboxylic acid. The combination of RjGlOx with *Agrobacterium* sp. DyP or *C. testosteroni* DyP generated new and enhanced amounts of low molecular weight aromatic products from organosolv lignin substrates, and was able to generate high-value products from treatment of lignin residue from cellulosic biofuel production, and from a polymeric humin substrate.

## Abstract 2

Lignin oxidation by bacterial dye-decolorizing peroxidase enzymes requires hydrogen peroxide as a co-substrate, an unstable and corrosive oxidant.

## Graphical abstract

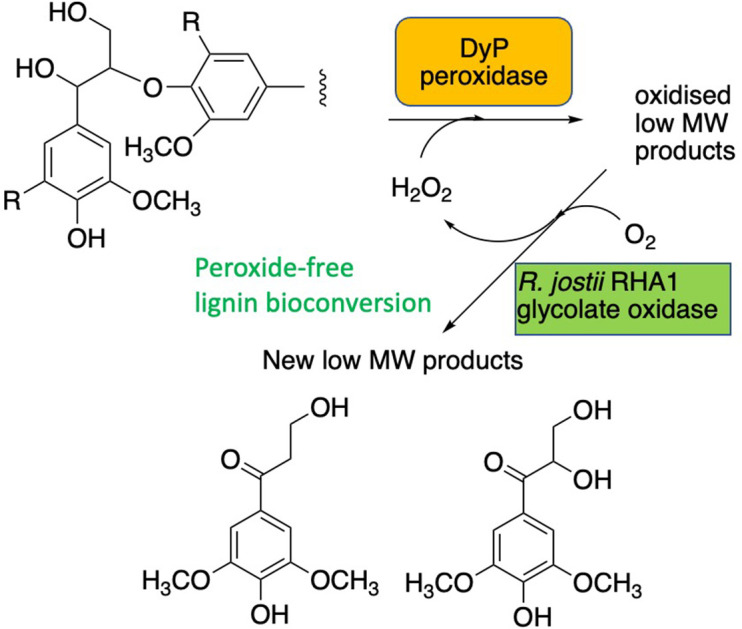

## Introduction

The conversion of aromatic biopolymer lignin to high-value products is a major unsolved problem in the lignocellulosic biorefinery concept, which is being studied around the world using a range of chemocatalytic and biocatalytic approaches.^1^ For biocatalytic lignin conversion, several classes of lignin-oxidising enzymes have been identified: from white-rot fungi, extracellular lignin peroxidase, manganese peroxidase and laccase enzymes have been identified for many years;^2,3^ in lignin-degrading soil bacteria, dye decolorizing peroxidases and multi-copper oxidase enzymes have also been identified in the last 10 years.^4,5^

However, although peroxidase enzymes offer considerable potential for lignin bio-conversion, there are practical problems in achieving successful bioconversions: principally due to repolymerisation of free radical intermediates in lignin oxidation; and inactivation of peroxidase enzymes by their hydrogen peroxide substrate.^4^ Several fungal oxidase enzymes have been characterised that can generate extracellular hydrogen peroxide,^6^ of which aryl alcohol oxidase^7^ and glyoxal oxidase^8^ appear to be closely linked with lignin breakdown. Aryl alcohol oxidase from *Pleurotus eryngii* is able to oxidise benzylic alcohols and aliphatic unsaturated alcohols efficiently,^9^ and this oxidation has been shown to generate hydrogen peroxide extracellularly in *Pleurotus eryngii*.^10^ The crystal structure of this enzyme has been determined,^11^ which has been used to guide protein engineering studies.^12^ Glyoxal oxidase from *Phanerochaete chrysosporium* is able to oxidise aliphatic aldehydes, α-hydroxycarbonyl compounds and α-dicarbonyl compounds,^13,14^ and has been shown to support hydrogen peroxide generation extracellularly in *P. chrysosporium.*^13^ Two further glyoxal oxidase enzymes have been characterised from *Pycnoporus cinnabarinus*.^15^

**Fig. 1:**
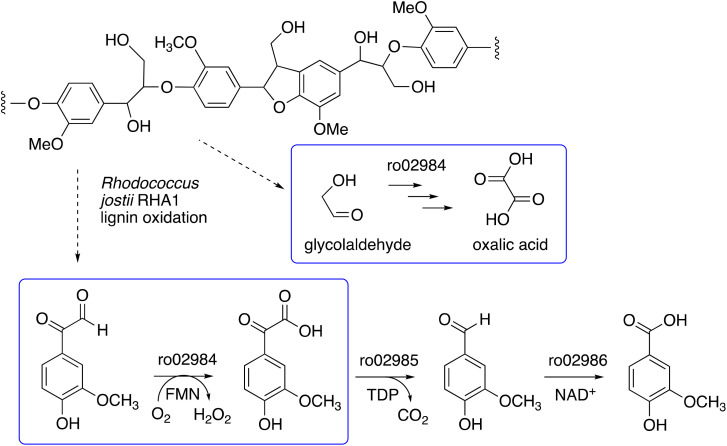
4-Hydroxybenzoylformate degradation pathway in *Rhodococcus jostii* RHA1, showing steps catalysed by glycolate oxidase (gene ro02984).^16,17^

We have recently identified a glycolate oxidase from lignin-degrading bacterium *Rhodococcus jostii* RHA1 as part of 4-hydroxybenzoylformate degradation gene cluster (gene ro02984, see Fig. 1) which is used to degrade aryl C_2_ fragments from lignin breakdown.^16^ Recombinant *R. jostii* glycolate oxidase is an FMN-dependent enzyme able to oxidise aromatic phenylglyoxal and mandelic acid substrates,^16^ and can catalyse successive oxidations of glycolaldehyde to glycolic acid, glyoxylic acid and oxalic acid.^17^*R. jostii* glycolate oxidase is not related to the fungal aryl alcohol oxidase or glyoxal oxidase families, but is a member of the vanillyl alcohol oxidase (VAO) superfamily that can oxidise 4-hydroxybenzyl alcohol substrates.^18^ Glycolate oxidase from spinach, which also utilises FMN, has been kinetically characterised,^19^ but bacterial glycolate oxidases have not been characterised biochemically. Since this enzyme generates hydrogen peroxide, we wished to investigate its substrate scope, and study whether it could act as an accessory enzyme for bacterial lignin degradation *in vitro*.

Although fungal aryl alcohol oxidase and glyoxal oxidase have been shown to support lignin generation *in vivo*,^9,12^ there is only one report of their use *in vitro* in combination with a peroxidase, where *P. eryngii* AAO has been used in combination with fungal lignin peroxidase.^20^ Since fungal lignin peroxidases are challenging to overexpress in recombinant form, there are potential advantages for a bacterial oxidase that could be combined with bacterial Dyp-type peroxidase enzymes, which can oxidise lignin.^4,5^ A copper-dependent oxidase from *Thermobifida fusca* has been reported to modify lignin in sugarcane bagasse,^21^ while pyranose 2-oxidase from *Kitasatospora aureofaciens* has also been reported to couple effectively with manganese peroxidase.^22^

Use of an oxidase-peroxidase combination would allow biotransformation of lignin using dioxygen as oxidant, rather than hydrogen peroxide. As well as using dioxygen as a milder oxidant, this would also avoid inactivation of the peroxidase enzyme by hydrogen peroxide. Use of biocatalysis for lignin biotransformation avoids the use of organic solvents and transition metals for chemocatalytic transformation of lignin,^1^ and avoids the use of energy-intensive high pressure hydrogenation^23^ or pyrolysis for lignin depolymerisation.^24^

Here we report that *R. jostii* glycolate oxidase can be used as an effective accessory enzyme for bacterial DyP peroxidase-catalysed peroxide-free oxidation of lignin. We also describe the activity of *R. jostii* glycolate oxidase for oxidation of hydroxymethylfurfural (HMF), a common by-product found in biorefinery lignins, and we report its application to valorisation of two biorefinery streams. Bioconversion of biomass-derived polymeric lignin to small molecule products offers a green chemistry solution to the production of chemicals from petroleum.^1^

## Experimental

### Materials

Chemicals & biochemicals were purchased from Sigma-Aldrich, except as indicated. Standards for HMF bioconversion were gifts from Dr Andrew Carnell (University of Liverpool). Poplar ammonia organosolv lignin,^25^ poplar alkaline organosolv lignin,^26^ and eucalyptus organosolv lignin^27^ were prepared as previously described,^25–27^ and were structurally characterised in Lancefield *et al.*^28^ The hydrolysis lignin (HL, supplied through the Zelcor project by Prof. S. Baumberger, INRAE Versailles) was produced from wheat straw by steam explosion pretreatment, followed by enzymatic hydrolysis, and was received as a wet cake (∼50 wt% water). Before use, the sample was dried at room temperature for 4 days, then manually milled and dried under vacuum to obtain a fine powder. HL contained 55 wt% lignin and 39 wt% carbohydrates by Klason assay, including 33.9 wt% cellulose and 4.8 wt% hemicelluloses.^29^ Humins were supplied by Avantium N. V. and were produced in their pilot plant in Geleen, The Netherlands, by conversion of fructose and glucose. Structural characterisation of these humins has been reported.^30^

Expression & purification of *Rhodococcus jostii* RHA1 glycolate oxidase (RjGlOx) from gene RHA1_ro02984 was described by Wei *et al.*^16^ Expression & purification of *Agrobacterium* sp. DyP peroxidase (AgroDyP) and *Comamonas testosteroni* DyP (CtDyP) was previously described by Rashid & Bugg.^31^

### Assay methods for *R. jostii* glycolate oxidase

#### Amplex Red assay

Kinetic assay for hydrogen peroxide production was carried out using the Amplex Red Hydrogen Peroxide/Peroxidase Assay kit (Invitrogen), according to manufacturer's instructions.^32^ Assays were carried out in 96-well microtitre plates in a HIDEX Sense microtiter plate reader, with fluorescence excitation and emission at 530 and 590 nm respectively. Glycolate solutions (0.5, 1, or 5 mM) with or without 0.1 mM FMN were prepared and incubated with an Amplex Red reagent/HRP working solution at room temperature, and fluorescence was immediately measured after 30 min.

#### Coupled enzyme assay

Kinetic parameters were measured *via* the H_2_O_2_-dependent oxidation of 2,2′-azinobis (3-ethylbenzthiazoline-6-sulfonic acid) (ABTS), using recombinant *P. fluorescens* DyP1B peroxidase to measure glycolate oxidase activity. Recombinant DyP1B was purified according to Rahmanpour *et al.*^33^ The reaction (total volume 1.0 mL) contained 200 μg DyP1B (9.6 mg mL^−1^ stock), 10 μM ABTS, 0.1–2 mM substrates and 90 μg glycolate oxidase enzyme (4.6 mg mL^−1^ stock) in 50 mM sodium phosphate buffer pH 6.0. The reaction was started by the addition of substrates, and the activity was followed at 30 °C for 2 min by measuring the absorbance at 420 nm (*ε*_420_ = 36 000 M^−1^ cm^−1^). All experiments were measured in triplicate, and substrate specificity was determined with the same assay. Michaelis–Menten plots with GraphPad Prism curve fitting software were used to calculate the kinetic parameters.

The optimum temperature was determined using glyoxal as substrate at 30, 42, 55, 60, and 70 °C. The pH optimum was measured in 50 mM glycine HCl buffer (pH 2.2–2.8), sodium acetate (pH 3–5), MES (pH 5.5–6.7) HEPES (pH 6.8–8.2), and MOPS (pH 6.5–7.9) buffers.

The thermal stability of the enzymes was determined by incubating the proteins at 30, 42, 55, 60, and 70 °C for 15 min, 30 min, 1 h, and 2 h. The enzyme was cooled in ice before measuring the activity.

#### HPLC assay

The products of the oxidation reaction of methylglyoxal, glyoxal, lactic acid, malic acid, benzaldehyde, 4-hydroxyphenylglyoxal, glycolaldehyde, glycolic acid, glyoxylic acid by glycolate oxidase was analysed by HPLC. Reaction mixtures were separated on an Aminex HPX-87H Organic Acids column (300 × 7.8 mm) (Bio-Rad) at 45 °C, with 5 mM sulfuric acid as mobile phase and a flow rate of 0.5 mL min^−1^. Eluted compounds were detected using UV at 254 nm. Reactions consisted of 50 mM sodium phosphate buffer pH 7.5 containing appropriate amount of enzyme, 2 mM substrate, and 0.1 mM FMN. The reactions were 45 incubated for 24 hours at 30 °C. Controls contained the same components except for enzyme. All reactions were filtered through 0.45 μm polyvinylidene difluoride syringe filters before injection in the column. Peak areas from the obtained chromatograms were converted to mass using calibration curves of pure substrates and products standards.

### Conversion of 5-hydroxymethylfurfural

#### In small scale

Reactions (total volume 1.0 mL) contained 2 mM (final concentration) 5-hydroxymethylfurfural, 0.1 mM FMN and 90 μg of glycolate oxidase (4.5 mg ml^−1^ stock) in 50 mM sodium phosphate buffer pH 7.5. Reactions were incubated for 24 hours at 30 °C. The conversion of 5-hydroxymethylfurfural to the corresponding products was analysed by HPLC using the Organic Acids HPX-87H column, as described above. Small scale conversion was also repeated in the presence of 40 μg bovine liver catalase (4 mg mL^−1^ stock).

#### In 200 mg scale

Reactions (total volume 100 mL) contained 200 mg (16 mM) 5-hydroxymethylfurfural, 0.1 mM FMN, and 9 mg glycolate oxidase (4.5 mg ml^−1^ stock) in 50 mM sodium phosphate buffer pH 7.5. Reactions were incubated for 24 hours at 30 °C. The conversion of 5-hydroxymethylfurfural to the corresponding products was analysed by HPLC using the Organic Acids HPX-87H column, as described above.

#### Conversion of lignin substrates by AgroDyP/CtDyP and RjGlOx

Colorimetric assays for aldehyde/ketone products using 2,4-dinitrophenylhydrazine, and phenolic products using Folin–Ciocalteu reagent, were carried out as previously described, in a 96-well microtitre plate reader.^31^

Conversions of 5 mg of lignin samples were carried out in a 1 mL total volume, containing 0.18 mg RjGIOx, 85 μg AgroDyp, 1 mM MnSO_4_, 0.1 mM FMN, 1 mM glycolic acid in 50 mM succinate buffer pH 6.5 at 30 °C. The mixture was left to incubate for 24 hours. A control incubation was set up without adding any enzymes. In order to perform LC/MS analysis, the material was acidified, and then extracted into ethyl acetate, dried *in vacuo*, and then reconstituted with 400 μL MeOH/water 1 : 1. A positive mode LC/MS analysis, with 270 nm monitoring, was used to examine the reaction components and compare them to authentic standards.

Biotransformation of 5 mg humin and futurol (dissolved in 200 μL DMSO) were carried out in a 1 mL total volume, containing 0.18 mg RjGlOx, 85 μg AgroDyp, 1 mM MnSO_4_, 0.1 mM FMN, 1 mM glycolic acid and 50 mM succinate buffer pH 6.5 at 30 °C. The mixture was left to incubate for 24 hours, and low molecular weight products extracted into ethyl acetate and examined by reverse phase C_18_ column by LC-MS.

#### LC-MS analysis

Samples for LC/MS analysis were extracted with ethyl acetate then solvent removed using a rotary vacuum evaporator. The organic residues were redissolved in 50% methanol/water. Samples were analysed on a reverse phase column hyperclone 5u BDS C18 column (130 Å, 250 mm, 4.6 mm) on Agilent 1260 infinity and Bruker amazon X mass spectrometer, at a flow rate of 0.5 mL min^−1^, monitoring at 270 nm. The solvent system was water (A) and methanol (B) containing 1% formic acid (for positive ionisation mode), gradient as follows: 5% B (0–5 min); 5–10% B (5–10 min); 10–75% B (10–35 min); 75–100% B (35–40 min); 100% MeOH (40–45 min); 100 to 10% B (45–50 min). MS parameters: Capillary 4500 V, end plate off set 500 V, Nebuliser pressure 25 psi, dry gas 8 L min^−1^, dry temperature 250 °C, mass range *m*/*z* 50–3000, target mass *m*/*z* 500, compound stability 100%, trap drive level 100%, ICC 200000.

## Results

### Biochemical characterisation of *Rhodococcus jostii* RHA1 GlOx

His_6_-tagged *R. jostii* glycolate oxidase was expressed and purified by immobilized metal affinity chromatography (see ESI Fig. S1†). The purified enzyme was yellow in colour, due to the FMN cofactor, with *λ*_max_ values at 375 and 456 nm (see ESI Fig. S2†).

**Fig. 2:**
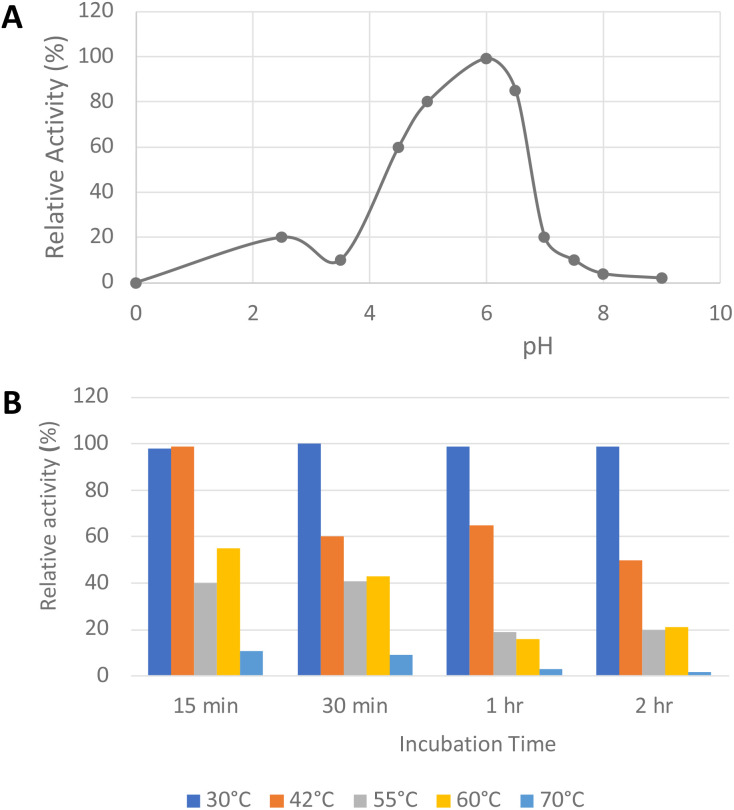
Activity of *R. jostii* glycolate oxidase *versus* pH (A) and temperature (B), using glyoxal as substrate, using coupled ABTS assay described in Experimental section.

The pH-rate profile of glycolate oxidase was determined *via* coupled enzyme assay, using recombinant *P. fluorescens* DyP1B peroxidase^33^ to couple the production of hydrogen peroxide with oxidation of 2,2′-azinobis (3-ethylbenz-thiazoline-6-sulfonic acid) (ABTS). The pH and temperature dependence of glycolate oxidase activity were measured with glyoxal as substrate, as shown in Fig. 2A. The enzyme showed optimal activity at pH 6.0, but showed >50% maximal activity in the range pH 4.5–6.5. The temperature dependence of the enzyme using glyoxal as substrate at pH 6.0 was measured at 30–70 °C, as shown in Fig. 2B. The enzyme retained activity at 30 °C or 42 °C, but lost activity upon incubation at 55 °C or above.

**Fig. 3:**
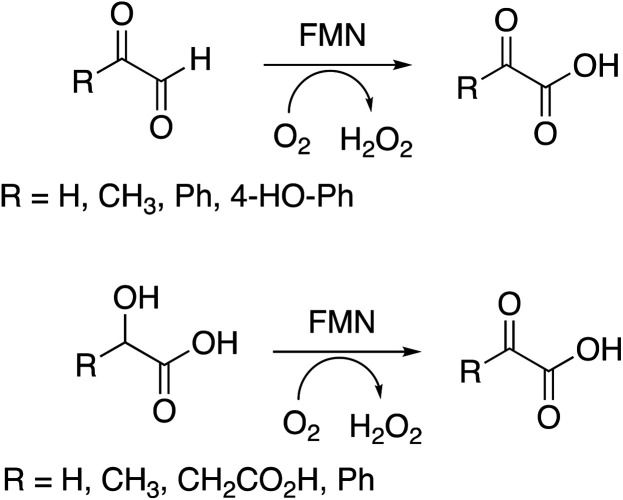
Substrates oxidised by *R. jostii* RHA1 glycolate oxidase.

Several substrates were tested for oxidation by RjGlOx, using the Dyp1B/ABTS coupled enzyme assay. As well as glycolic acid, RjGlOx was able to oxidise α-hydroxy acids l-lactic acid, l-malic acid, and mandelic acid (see Fig. 3). It was also able to oxidise α-ketoaldehyde substrates glyoxal, methylglyoxal, phenylglyoxal, and 4-hydroxyphenylglyoxal. However, no activity was observed for benzaldehyde, hence the enzyme requires the presence of two adjacent functional groups in the substrate in order to show oxidation activity. Michaelis–Menten steady-state kinetic parameters were measured, as shown in Table 1 (kinetic plots shown in ESI Fig. S3†). The products obtained from RjGlOx conversion of each substrate were verified by HPLC analysis, as noted in Table 1 (see ESI Fig. S4†). Similar activities were observed for several α-dicarbonyl and α-hydroxycarboxylic acid substrates, hence this enzyme appears to show rather broad substrate specificity. In an earlier study of lignin metabolites observed in *Rhodococcus jostii* RHA1, we also observed that this enzyme can catalyse benzylic oxidation of 4-hydroxyphenylacetic acid to 4-hydroxymandelic acid and 4-hydroxybenzoylformic acid.^17^

**Table 1:** Kinetic parameters for oxidation of α-dicarbonyl and α-hydroxycarboxylic acid substrates by *R. jostii* RHA1 glycolate oxidase. See Fig. 3 for chemical structures. Steady-state kinetic plots are shown in ESI Fig. S3†

Substrate	Product	Specific activity (μmol min^−1^ mg^−1^)	*K*_M_ (mM)	*k*_cat_ (s^−1^)	*k*_cat_/*K*_M_ (M^−1^ s^−1^)
Glyoxal (R = H)	Oxalic acid^a^	0.45	1.92	0.44	230
Methylglyoxal (R = CH_3_)	Pyruvic acid^a^	0.97	0.43	0.88	2050
4-Hydroxyphenylglyoxal	4-Hydroxybenzoylformate^a^	0.21	0.25	0.21	830
Glycolaldehyde	Glycolic acid^b^	0.49	0.69	0.48	700

Glycolic acid (R = H)	Glyoxylic acid^b^	0.78	0.49	0.77	1560
L-Lactic acid (R = CH_3_)	Pyruvic acid^a^	0.48	0.24	0.47	1970
Malic acid (R = CH_2_CO_2_H)	Oxaloacetic acid^a^	0.29	0.29	0.60	2060
Mandelic acid (R = Ph)	Benzoylformic acid^a^	1.92	0.50	1.90	3800
Glyoxylic acid	Oxalic acid^b^	0.55	0.64	0.62	970

a Product identification by HPLC shown in ESI Fig. S4.†

b Product identification shown previously.^17^

### Activity of RjGlOx for oxidation of 5-hydroxymethylfurfural to 2,5-furandicarboxylic acid

**Fig. 4:**
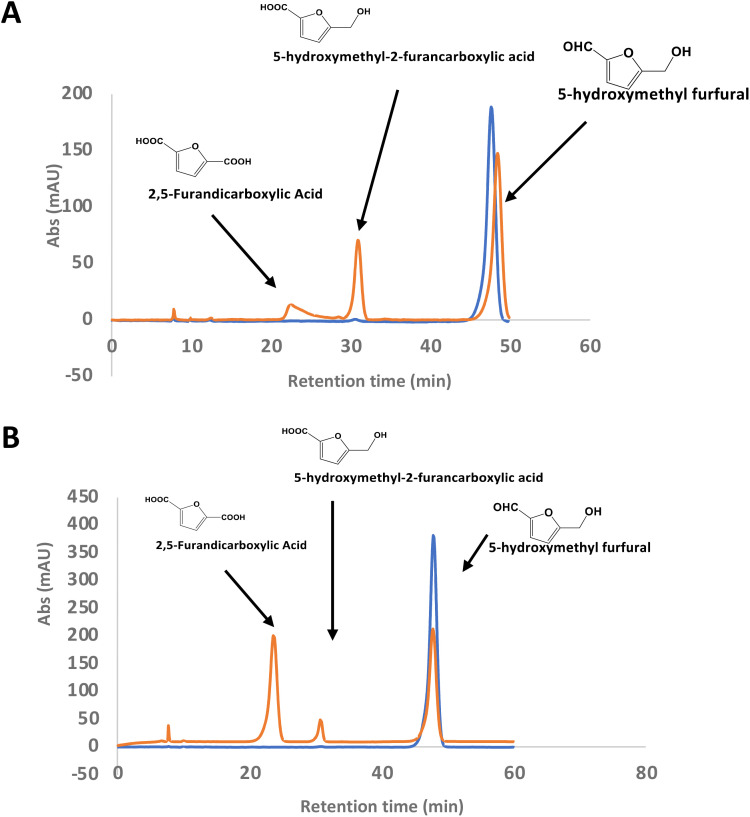
Conversion of HMF to FDCA by *R. jostii* RHA1 glycolate oxidase. (A) Small scale (1 ml) conversion of 2 mM HMF by 90 μg Rj GlOx; (B) large scale (100 ml) conversion of 200 mg (16 mM) HMF by 9 mg Rj GlOx. Orange, reaction mixture containing enzyme; blue, control reaction lacking enzyme. All bioconversions incubated in 50 mM phosphate buffer pH 7.5 for 24 h at 30 °C.

There is considerable interest in the oxidation of 5-hydroxymethylfurfural (HMF) to 2,5-furandicarboxylic acid (FDCA), since FDCA can be used to generate polyester bioplastics.^34^ Enzyme-catalysed oxidation of HMF to FDCA has been reported by HMF oxidase from *Methylovorus* sp. MP688,^35,36^ and a combination of oxidase PaoABC with glucose oxidase variants,^37^ however, both systems show modest rates of turnover to FDCA. A further oxidase from *Moesziomyces antarcticus* has been reported to convert HMF to FFCA, and a further unspecific peroxygenase used to convert FFCA to FDCA.^38^ Since *R. jostii* glycolate oxidase shows activity for oxidation of bifunctional aldehyde substrates, we tested HMF as a substrate for RjGlOx-catalysed oxidation. We observed 20–30% conversion on a small scale of 2 mM HMF to 5-hydroxymethyl-2-furancarboxylic acid (HMFCA) and FDCA (see Fig. 4A), indicating that the aldehyde sidechain is oxidised first to a carboxylic acid, followed by oxidation of the hydroxymethyl group. On a 200 mg scale, conversion of 16 mM HMF with addition of 9 mg RjGlOx in 50 mM phosphate buffer pH 7.5 gave FDCA as the major product peak in 45–50% yield, but with residual HMF substrate, and some HMFCA (see Fig. 4B). Addition of 40 μg bovine liver catalase on a small-scale conversion of 2 mM HMF was observed to improve the bioconversion of HMF (see ESI Fig. S5†), but under these conditions the major product was HMFCA, with a 23% conversion to FDCA. *R. jostii* glycolate oxidase is therefore a new biocatalyst for HMF oxidation, which although it has lower catalytic efficiencies than some reported oxidases,^37,38^ can oxidise HMF to FDCA in a single enzyme biotransformation, and can be expressed efficiently in *Escherichia coli*.

### Coupling of RjGlOx with AgroDyp and CtDyP

Since RjGlOx generates hydrogen peroxide, it could potentially act as an accessory enzyme for peroxidase-catalysed oxidation of lignin. We have previously identified bacterial dye-decolorizing peroxidases DypB from *Rhodococcus jostii* RHA1^39^ and Dyp1B from *Pseudomonas fluorescens* pf-5^33^ with activity for oxidation of polymeric lignin, however, in common with most peroxidases, these enzymes are most active at pH 4–5. We have recently identified two further bacterial dye decolorizing peroxidases from *Agrobacterium* sp. and *Comamonas testosteroni* that retain significant activity at pH 6–7.^31^ Comparing their pH-rate profiles with *R. jostii* glycolate oxidase (see ESI Fig. S6†), we found that all three enzymes retained >80% maximum activity at pH 6.5. Therefore we tested RjGlOx in combination with AgroDyP or CtDyP at pH 6.5, using different polymeric lignins.

**Fig. 5:**
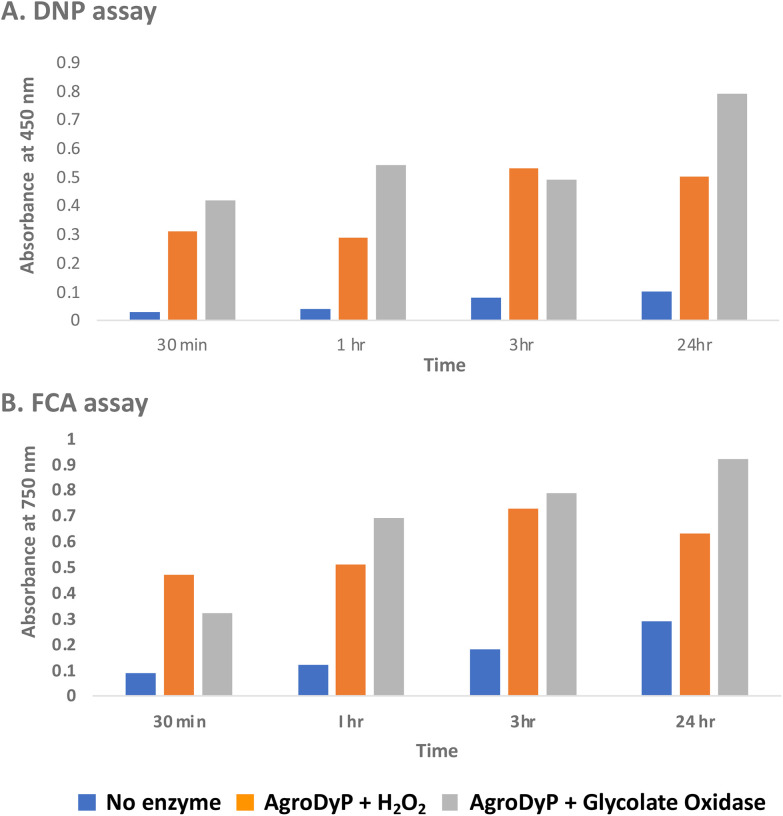
Formation of low molecular weight ketone/aldehyde products *via* 2,4-dinitrophenylhydrazine assay (A) and phenolic products *via* Folin–Ciocalteu assay (B) from conversion of poplar alkaline organosolv lignin by *Agrobacterium* DyP/1 mM hydrogen peroxide (in orange) *vs. Agrobacterium* DyP/*R. jostii* glycolate oxidase/1 mM glycolic acid (in grey). Assay data for conversion of other polymeric lignins are shown in ESI.†

We first tested for the formation of low molecular weight products from polymeric lignin, using two published colorimetric assays, using 2,4-dinitrophenylhydrazine to derivatise ketone and aldehyde products, and the Folin–Ciocalteu assay to measure low molecular weight phenol products,^31^ as shown in Fig. 5. Four different samples of polymeric were used, that were characterised in a previous study:^28^ poplar alkaline and ammonia organosolv lignin, eucalyptus organosolv lignin, and a miscanthus ionic liquid lignin. Activity of *Agrobacterium* DyP in the presence of 1 mM hydrogen peroxide was compared with AgroDyP and RjGlOx in the presence of 1 mM glycolic acid. As shown in Fig. 5A, using the DNP assay, the conversion with AgroDyP/RjGlOx in the absence of hydrogen peroxide in fact generated more low molecular weight products than AgroDyP/H_2_O_2_: it showed 30% higher activity after 30 min, and remained active over 24 h, whereas in the presence of hydrogen peroxide, AgroDyP lost activity after 3 h. Using the FCA assay, for AgroDyP/H_2_O_2_, product formation after 24 h was reduced, consistent with some repolymerisation of phenolic products, but this was not observed for the peroxide-free conversion, suggesting that the coupled RjGlOx conversion may also reduce the degree of repolymerisation. Similar trends were observed using other polymeric lignin substrates (see ESI Fig. S7 and S8†).

**Fig. 6:**
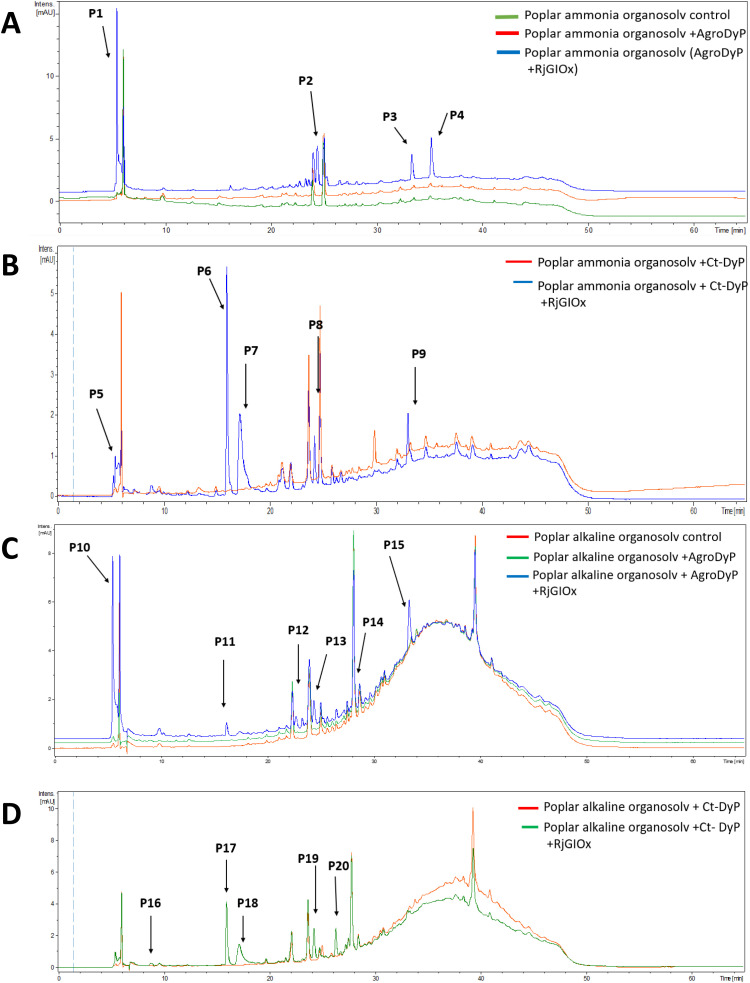
Total ion chromatograms for product formation from combined DyP/GlOx transformations of polymeric lignin substrates. Bioconversion of poplar ammonia organosolv lignin by AgroDyp/RjGlOx (A) and CtDyp/RjGlOx (B), and conversion of poplar alkaline organosolv lignin by AgroDyp/RjGlOx (C) and CtDyp/RjGlOx (D), are compared with control incubations containing DyP peroxidase alone, or no enzyme. Peaks that show increased intensity or are absent without RjGlOx are indicated, and they are identified in Table 2.

In order to investigate whether the product distribution had changed in the presence of *R. jostii* glycolate oxidase, the low molecular weight products were extracted and analysed by LC-MS, using poplar ammonia organosolv lignin, poplar alkaline organosolv lignin, and eucalyptus organosolv lignin, which had been shown previously to generate low molecular weight products using *Pseudomonas fluorescens* Dyp1B.^28^ Analysis of transformations with AgroDyp or CtDyP in the presence or absence of RjGlOx showed the formation of new product peaks, and enhanced peak heights for existing peaks, in the presence of RjGlOx (see Fig. 6), indicating that the combined DyP/GlOx bioconversion generates increased product yield and some new reaction products, compared with DyP alone. Although there are few published studies on lignin bioconversion with enzyme combinations, we observed previously the formation of new products from lignin bioconversion with DyP peroxidases and other accessory enzymes.^31^

**Table 2:** Products with increased yield or absent without RjGlOx. Products were identified by comparison with authentic standards where available. LC-MS traces are shown in ESI Fig. S9;† estimated product concentrations shown in ESI Fig. S10;† standards shown in ESI Fig. S11.† Products P16 and P3/P9/P15 have been observed in previous transformations of polymeric lignin by *P. fluorescens* Dyp1B.^26^ Chemical structures of aromatic products are illustrated in the Discussion section. ND, not determined

		Poplar ammonia organosolv lignin		Poplar alkaline organosolv lignin		
RT (min)	*m*/*z*	AgroDyp/RjGlOx	CtDyp/RjGlOx	AgroDyp/RjGlOx	CtDyp/RjGlOx	Product identity
5.2	228		P5			ND
5.5	99	P1		P10		Glyoxylic acid
8.7	157				P16	2-Methoxy-6-hydroxy-1,4-hydroquinone
16.0	125		P6	P11	P17	Guaiacol
17.2	153		P7		P18	Vanillin
22.0	ND			P12		ND
24.3	155		P8	P13	P19	Protocatechuic acid
24.5	169	P2				Vanillic acid
26.3	183				P20	Syringaldehyde
29.0	146			P14		ND
33.1	243	P3	P9	P15		Syringyl 1-keto-2,3-dihydroxypropane
35.2	582	P4				Unidentified oligomer

As shown in Table 1, a number of products were identified by LC-MS analysis. Many of these products have been observed in previous lignin bioconversions,^28,31^ and their formation will be discussed further in the Discussion section. Products guaiacol and 2-methoxy-6-hydroxy-1,4-hydroquinone arise from aryl-C_α_ bond cleavage, whereas vanillin, syringaldehyde, vanillic acid and protocatechuic acid arise from C_α_–C_β_ bond cleavage. Ct-DyP showed a stronger preference to generate guaiacol and vanillin products. A syringyl monomer containing an oxidised keto-diol sidechain was observed in the presence of RjGlOx, which could have arisen from sidechain oxidation by glycolate oxidase. Glyoxylic acid was also observed as a product, which can be generated by RjGlOx-catalysed oxidation of glycolaldehyde.^17^ Further LC-MS data for transformation of eucalyptus organosolv lignin is shown in ESI (Fig. S12†), in which several of the same products were also generated, and the case of CtDyP/RjGlOx, syringic acid was also observed as a product.

### Application to biorefinery streams

In the lignocellulose-based biorefinery concept, pretreatment of lignocellulosic biomass improves for availability of cellulosic sugars for saccharification, however, a practical problem with pretreatment is the formation of fermentation inhibitors and enzyme inhibitors,^40^ notably aldehydes such as 5-hydroxymethylfuran (HMF) and furfural,^41^ which limits the utilisation of both cellulose and lignin streams. Since RjGlOx shows activity for oxidation of HMF and other aldehydes, we investigated whether the combination of DyP peroxidases and RjGlOx would improve the generation of high-value chemicals from biorefinery streams arising from lignocellulose pretreatment.

**Fig. 7:**
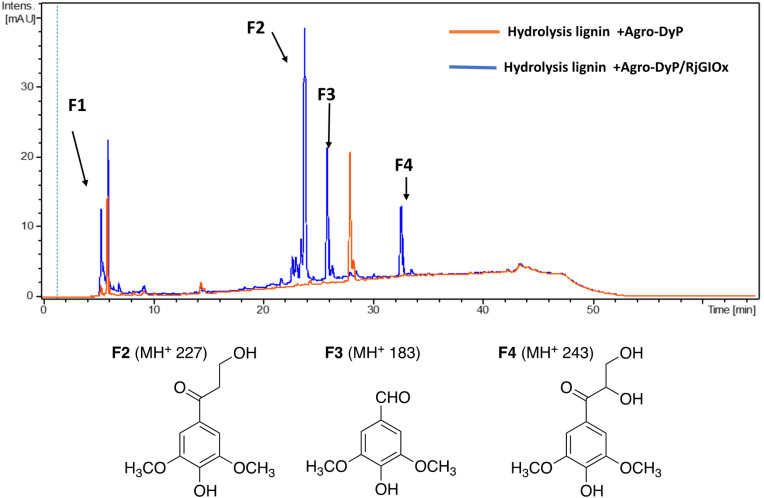
Total ion chromatogram of products from bioconversion of hydrolysis lignin by AgroDyP and RjGlOx, and chemical structures of identified products.

A combination of AgroDyP and RjGlOx was applied to bioconversion of two biorefinery streams: hydrolysis lignin (HL) produced after pretreatment and enzymatic saccharification of wheat straw for cellulosic bioethanol production;^29^ and humins, polymeric materials derived from polymerisation of HMF.^30^ In the case of HL, 4 new peaks were observed in the presence of the AgroDyP/RjGlOx combination, as shown in Fig. 7. Peak F3 was identified as syringaldehyde (*m*/*z* 183 MH^+^), by comparison with an authentic standard, and has been observed in previous lignin bioconversions.^28^ Peak F4 (33.0 min, *m*/*z* 243) was also observed in lignin bioconversions above (peak P3 in Table 1), and is proposed to be syringyl 2,3-dihydroxypropan-1-one, a monomer containing a keto-diol C_3_ sidechain. A syringyl monomer containing a C_3_ triol sidechain has been observed in a previous lignin bioconversion,^28^ and a keto-diol sidechain has been observed in a lignin dimer product arising from lignocellulose oxidase by Dyp1B.^33^ Peak F2 (24.2 min, *m*/*z* 227) is proposed to be syringyl 3-hydroxypropan-1-one, for which a related guaiacyl product has been observed previously in a DyP peroxidase biotransformation.^28^ Peak F1 (5.2 min, *m*/*z* 228) is an unknown product.

**Fig. 8:**
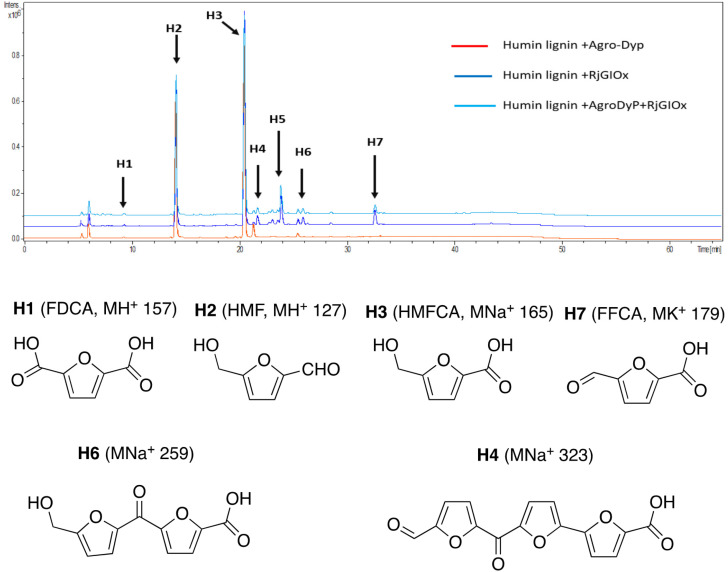
Total ion chromatogram of products from bioconversion of Avantium humins by AgroDyP and RjGlOx. Structures for H1–H3 and H7 confirmed by co-elution with authentic standards, structures for H4 and H6 based on predicted humin structure.^30^

In the case of Avantium humins, 7 new peaks were observed with the AgroDyp/RjGlOx combination, as shown in Fig. 8. Peak H2 was identified as 5-hydroxymethylfurfural (HMF), which was also observed in much smaller amounts (<10%) in a control incubation lacking enzyme. 5-Hydroxymethylfurfural (peak H2) and 5-hydroxymethyl-2-furancarboxylic acid (HMFCA, peak H3) were produced as major products, while further compounds related to HMF were identified by comparison with authentic standards, namely 2,5-furandicarboxylic acid (FDCA, peak H1) and 5-formyl-2-furancarboxylic acid (FFCA, peak H7). Based on the proposed structure for polymeric humins,^30^ and the observed activities of RjGlOx, we have proposed feasible hypothetical dimeric and trimeric structures in Fig. 7 for peaks H6 (25.5 min, *m*/*z* 259) and H4 (21.8 min, *m*/*z* 323), while peak H5 (24.0 min, *m*/*z* 457) appears to be a tetrameric furanic compound.

## Discussion

*Rhodococcus jostii* RHA1 glycolate oxidase is shown to be a valuable accessory enzyme for degradation of polymeric lignin *in vitro*. Although first identified as an oxidase for generation of aromatic benzoylformate pathway intermediates,^16^ we show that it has a broad substrate scope for α-ketoaldehyde and α-ketoacid substrates, and for oxidation of HMF to 2,5-FDCA. The kinetic parameters measured in Table 1 indicate that highest *k*_cat_ and *k*_cat_/*K*_M_ were observed for mandelic acid, but for dicarbonyl substrates, methylglyoxal has a higher *k*_cat_ and *k*_cat_/*K*_M_ than 4-hydroxyphenylglyoxal. α-Substituted aldehydes such as glycolaldehyde and methylglyoxal are known by-products of biomass conversion,^42^ therefore, this enzyme could oxidise these aliphatic aldehydes during lignin oxidation, and we have already shown that this enzyme oxidises glycolaldehyde to oxalic acid during lignin breakdown.^17^ The *R. jostii* RHA1 glycolate oxidase gene ro02984 contains no signal sequence, therefore it is unlikely that this enzyme is used to generate hydrogen peroxide extracellularly, however, it could participate in the oxidation of lignin fragments intracellularly. We note that overexpression of *Pseudomonas putida* KT2440 glycolate oxidase genes *glcDEF* has been recently reported to permit growth on ethylene glycol, catalysing the removal of toxic intermediates.^43^ On the basis of the experimental data, there does not seem to be one preferred substrate for *R. jostii* glycolate oxidase, we therefore suspect that it has a dual role in the cell (as shown in Fig. 1), for oxidation of aryl-C_2_ substrates to be processed by the 4-hydroxybenzoylformate pathway,^16^ but also to oxidise glycolaldehyde and methylglyoxal generated from lignin degradation, which would otherwise be toxic to the cell.

**Fig. 9:**
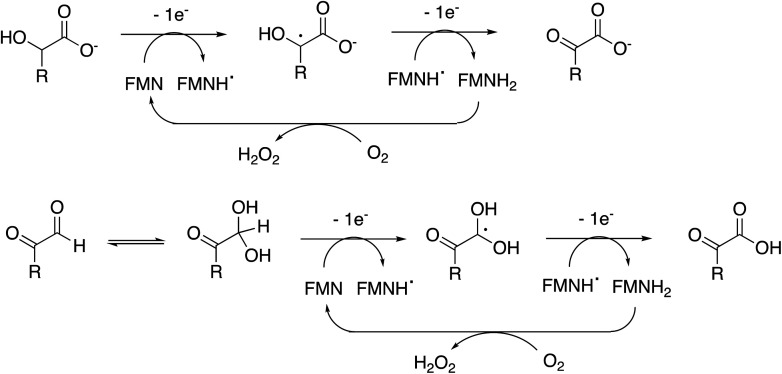
Proposed catalytic mechanism for RjGlOx-catalysed oxidation of α-hydroxy-carboxylic acid and α-dicarbonyl substrates. R can be either an aromatic or aliphatic (H, –CH_3_, –CH_2_CO_2_H) group.

The enzyme requires two functional groups to be present for oxidation to take place, which may be a consequence of the enzyme mechanism. We have previously observed conversion of 4-hydroxyphenylacetic acid to 4-hydroxybenzoylformate, *via* hydroxylation of benzylic position.^17^ The likely mechanism for this reaction involves hydrogen atom abstraction (or deprotonation followed by 1-electron transfer) in the benzylic position, *via* a radical intermediate. If a similar hydrogen atom abstraction mechanism applied to aldehyde and α-hydroxy-acid oxidation, as shown in Fig. 9, then this would rationalise why two functional groups are needed, since the radical intermediate would be stabilised by adjacent carboxylic acid (or ketone) group. In the case of HMF oxidation, a radical intermediate would be stabilised by the adjacent furan ring.

RjGlOx co-operates effectively with DyP peroxidases AgroDyP and CtDyp *in vitro*, since they retain activity at pH 6.5, to enable a bioconversion driven by dioxygen as an oxidant, rather than hydrogen peroxide. This is a milder, more sustainable process than using a peroxidase with added hydrogen peroxide, which also leads to less enzyme inactivation (shown in data in Fig. 5). Although some hydrogen peroxide will be generated during the biotransformation by RjGlOx, it will likely be sub-millimolar concentrations, since *K*_M_ for hydrogen peroxide by DyP peroxidases is in the range 50–70 μM,^33^ and *k*_cat_ values for DyPs are in the range 10–20 s^−1^,^33^ compared with 0.5–2 s^−1^ for RjGlOx (see Table 1). Since inactivation of DyPs occurs in the presence of 1–2 mM hydrogen peroxide,^39^ it is logical that controlled release of hydrogen peroxide should be beneficial.

**Fig. 10:**
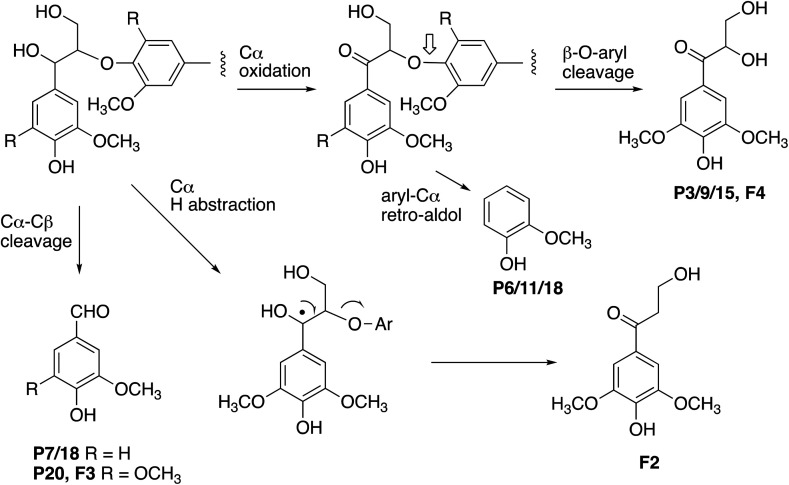
Formation of products from polymeric lignin. R = H (guaiacyl G lignin unit) or OCH_3_ (syringyl S lignin unit). Further oxidation of vanillin (P7/18) generates the corresponding carboxylic acid, vanillic acid (P2). Poplar organosolv lignin contains both S and G units (with proportion of S > G).

The formation of higher product yields from polymeric lignin oxidation (see Fig. 7–9) is most likely due to a more effective DyP oxidation, since there is less enzyme inactivation, but there is a possibility that RjGlOx might also prevent repolymerisation of radical products, *via* further 1-electron oxidation, or 1-electron reduction by a flavin semiquinone intermediate. We have shown previously that combination of DyP peroxidases with other accessory enzymes that prevent lignin repolymerisation can lead to enhanced yield of products from polymeric lignin substrates.^31^ The formation of alternative products may be aided by RjGlOx-catalysed oxidation reactions: two new products (ketodiol P3/9/15 and glyoxylic acid P1/10) contain carbonyl groups that could arise from GlOx-catalysed oxidation. The formation of the various reaction products is rationalised in Fig. 10: C_α_–C_β_ oxidative cleavage is precedented for DyP peroxidases;^33,39^ the formation of ketodiol P3/9/15 and guaiacol P6/11/17 seems most likely *via* a benzylic ketone intermediate; and ketoalcohol product F2 could perhaps be formed *via* elimination of the β-*O*-aryl substituent *via* a benzylic radical, which might be catalysed by RjGlOx on an oligomeric lignin fragment. The formation of a reaction product containing an oxidised ketodiol sidechain has also been observed previously from lignocellulose, by *P. fluorescens* Dyp1B.^33^

One interesting point is that for the combined DyP/GlOx conversion of polymeric lignin, each enzyme provides the substrate for the other, since GlOx provides hydrogen peroxide for DyP, but also DyP generates oxidised small molecule products, such as aldehydes, from lignin breakdown. This is seen in the formation of ketodiol product P3/9/15 from organosolv lignin. Hence there is a kind of symbiotic relationship between these two enzymes.

**Fig. 11:**
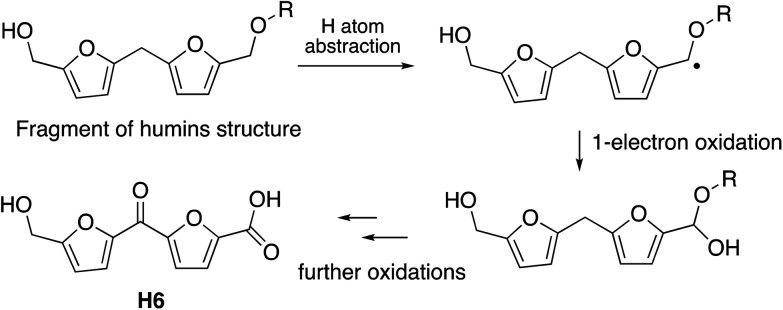
Possible mechanism for generation of product H6 from oxidation of humins *via* combined RjGlOx/DyP biotransformation. A similar mechanism can be drawn for the formation of product H4.

The observation that the RjGlOx/DyP combination is effective for oxidation of biorefinery streams which contain high levels of aldehydes could lead to new applications for generation of high value chemicals from a lignocellulosic biorefinery. Reaction products F4 from HL and H4 and H6 from bioconversion of humins each contain ketone groups which could arise from GlOx oxidation. The low background in the case of HL implies that GlOx actively participates in product release, whereas in the case of humins, some products are released by GlOx alone (see Fig. 8). Although we do not know the precise mechanism for oxidation of humins, it is possible that GlOx could catalyse oxidation of an ether-linked unit found in humins, as shown in Fig. 11, leading to the formation of an aldehyde sidechain, which could be further oxidised to a carboxylic acid, and the methylene unit also oxidised to a ketone. Hence we see new products and new mechanisms through the use of enzyme combinations, which offer new possibilities for oxidation of polymeric lignin/humins *in vitro*.^31^

## Author contributions

The research was carried out by AA and GMMR. The project was supervised by TDHB. The funding for the work was obtained by TDHB and AA. The first draft of the manuscript was written by TDHB and AA.

## Conflicts of interest

The authors declare no conflict of interest associated with this article.

## Supplementary Material

GC-025-D3GC00475A-s001
