# Supplementary material for: Application of Rhodococcus jostii RHA1 glycolate oxidase as an efficient accessory enzyme for lignin conversion by bacterial Dyp peroxidase enzymes
Source: Green Chem. 2023 Apr 11;25(9):3549–60. doi: 10.1039/d3gc00475a (PMC10167727; doi:10.1039/d3gc00475a)
Supplement: GC-025-D3GC00475A-s001 [file GC-025-D3GC00475A-s001.pdf]

Electronic Supplementary Material (ESI) for Green Chemistry.
This journal is © The Royal Society of Chemistry 2023

Application of *Rhodococcus jostii* RHA1 glycolate oxidase as an efficient accessory enzyme for lignin conversion by bacterial Dyp peroxidase enzymes

Awatif Alruwaili, Goran M.M. Rashid, and Timothy D.H. Bugg*

Supporting Information

Figure S1. Purification of recombinant *R. jostii* RHA glycolate oxidase

Figure S2. UV-visible spectrum of purified recombinant *R. jostii* glycolate oxidase

Figure S3. Michaelis-Menten steady-state kinetic plots for conversion of α-dicarbonyl and α-hydroxycarboxylic acid substrates by recombinant *R. jostii* RHA1 glycolate oxidase.

Figure S4. Analysis of reaction products of *R. jostii* RHA1 glycolate oxidase transformations by HPLC.

Figure S5. Conversion of HMF to FDCA by *R. jostii* RHA1 glycolate oxidase in the presence of catalase

Figure S6. Activity vs pH for *Agrobacterium* DyP and *Comamonas testosteroni* DyP compared with *R. jostii* glycolate oxidase

Figure S7,S8. Assays of low molecular weight ketone/aldehyde formation from polymeric lignins using 2,4-dinitrophenylhydrazine (S7) and Folin-Ciocalteu reagent (S8).

Figure S9. Extracted ion chromatograms for product peaks P2-P20 from conversion of poplar ammonia organosolv lignin or poplar alkaline organosolv lignin by Agro-Dyp/Ct-DyP and RjGlOx

Figure S10,S11. Estimated concentrations of reaction products from bioconversion of polymeric lignin and humins (S10) by comparison with authentic standards (S11).

Figure S12. Total ion chromatograms for bioconversion of eucalyptus organosolv lignin by (A) Agro-DyP and RjGlOx (B) Ct-DyP and RjGlOx (C) extracted ion chromatogram for new product peak P31.

Figure S13. Extracted ion chromatograms for product peaks from incubation of hydrolysis lignin (HL) with Agro-Dyp enzyme in the presence of RjGIOx.

Figure S14. Extracted ion chromatograms for product peaks from incubation of humins with Agro-Dyp enzyme in the presence of RjGIOx.

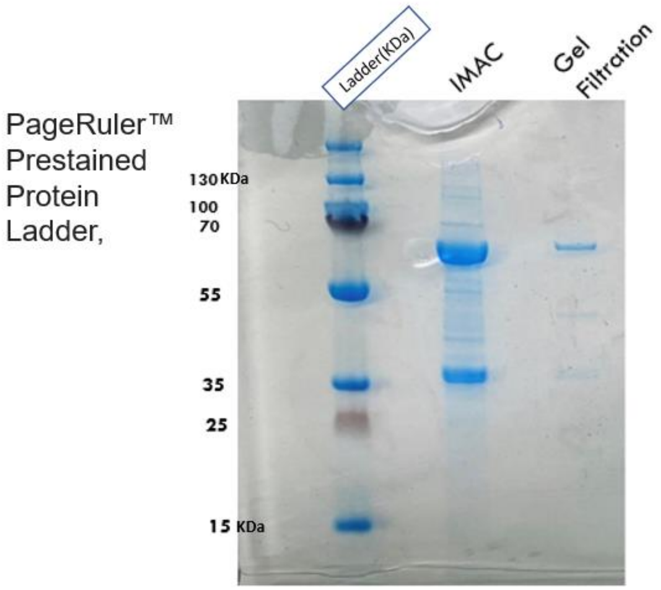

Figure S1. Purification of recombinant *R. jostii* RHA glycolate oxidase by immobilized metal affinity chromatography, followed by PD10 gel filtration. The lower band at 35 kDa was found by proteomic analysis to be a smaller fragment of the same protein.

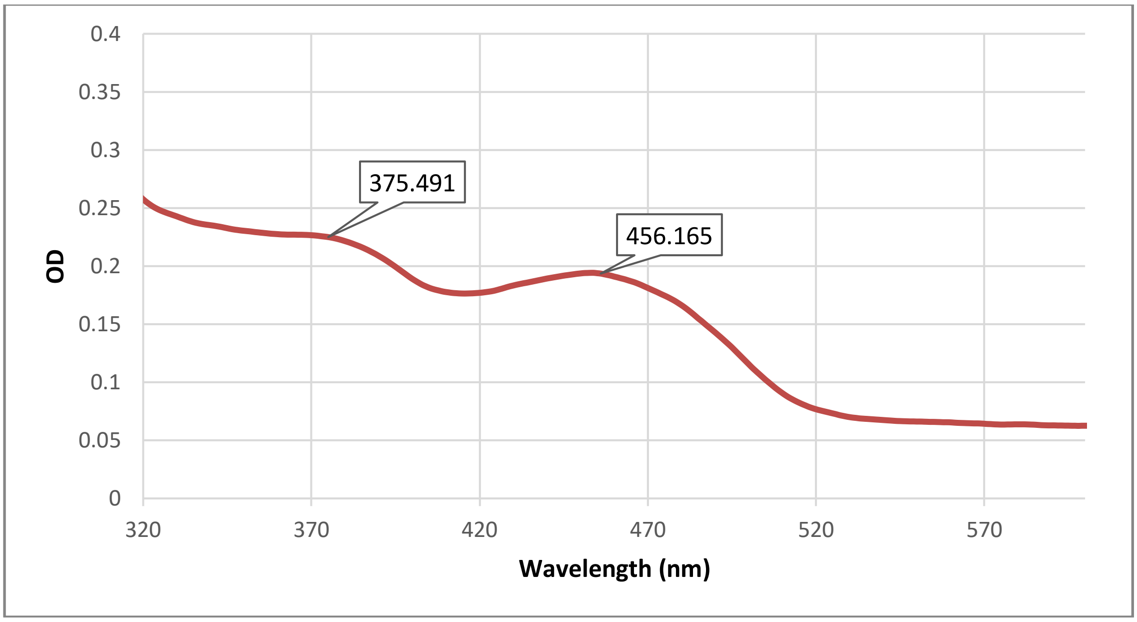

Figure S2. UV-visible spectrum of 4.6 mg/ml purified recombinant *R. jostii* glycolate oxidase in 50 mM phosphate buffer pH 7.5.

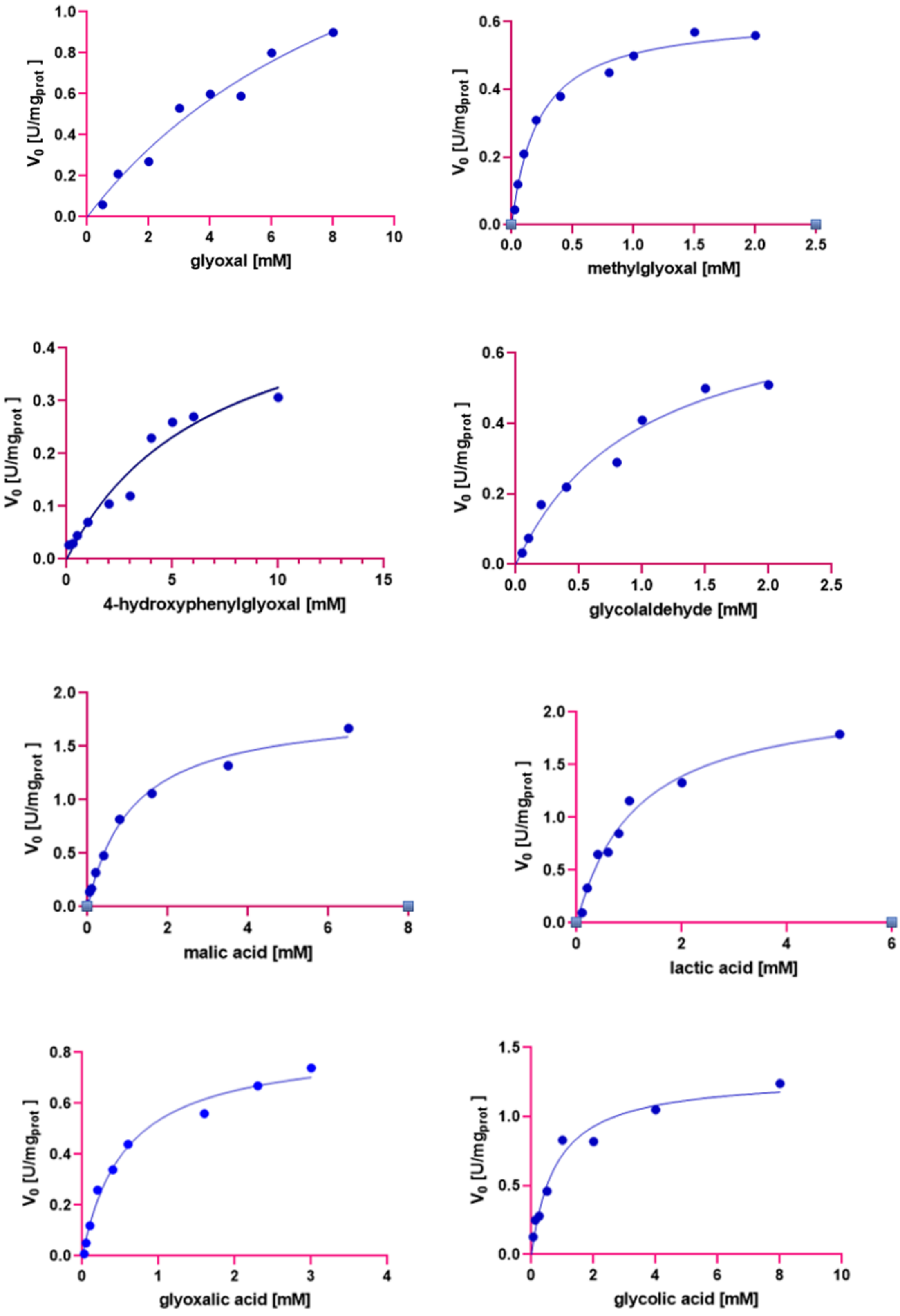

Figure S3. Michaelis-Menten steady-state kinetic plots for conversion of α-dicarbonyl and α-hydroxycarboxylic acid substrates by recombinant *R. jostii* RHA1 glycolate oxidase.

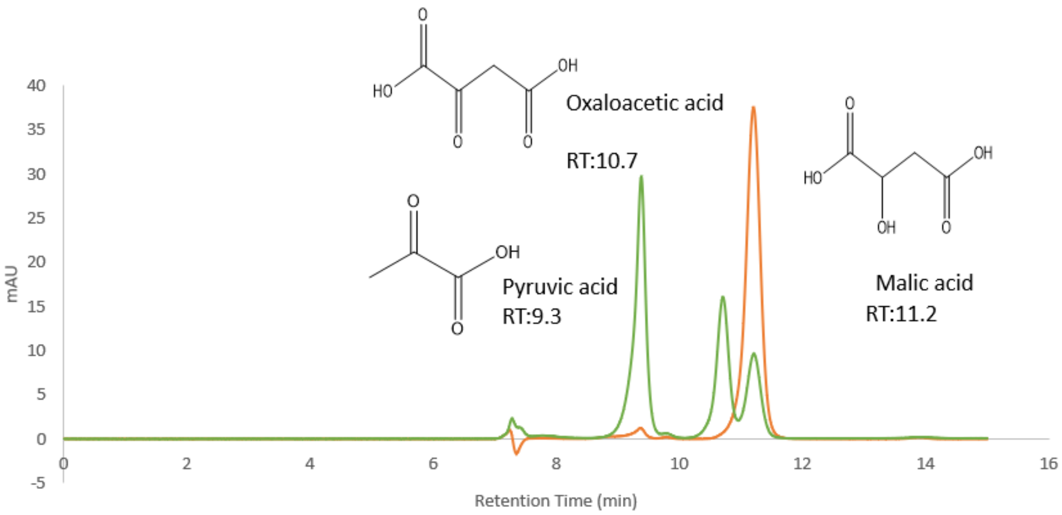

Figure S4A. HPLC analysis of reaction products from incubation of malic acid with *Rhodococcus jostii* RHA1 glycolate oxidase enzyme. Green line, sample treated with *R. jostii* RHA1 glycolate oxidase; orange line, control lacking glycolate oxidase enzyme. Aminex HPX-87H Organic Acids column (Bio-Rad) at 45°C, eluent 5 mM sulfuric acid flow rate 0.5 mL/min.

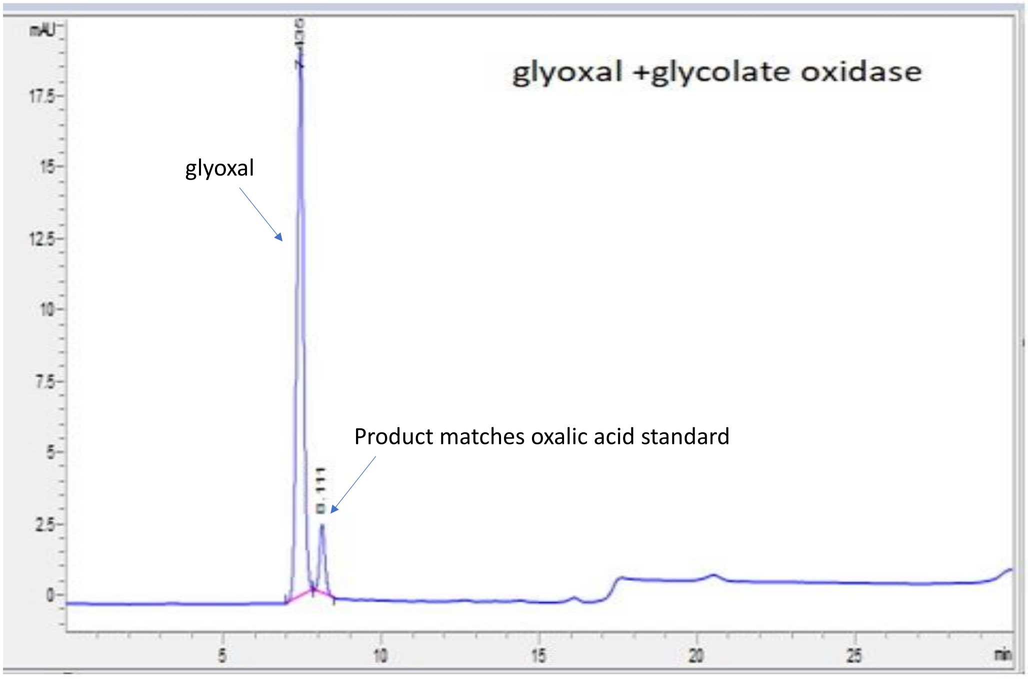

Figure S4B. HPLC analysis of reaction products from incubation of glyoxal with *Rhodococcus jostii* RHA1 glycolate oxidase enzyme. Aminex HPX-87H Organic Acids column (Bio-Rad) at 45°C, eluent 5 mM sulfuric acid flow rate 0.5 mL/min.

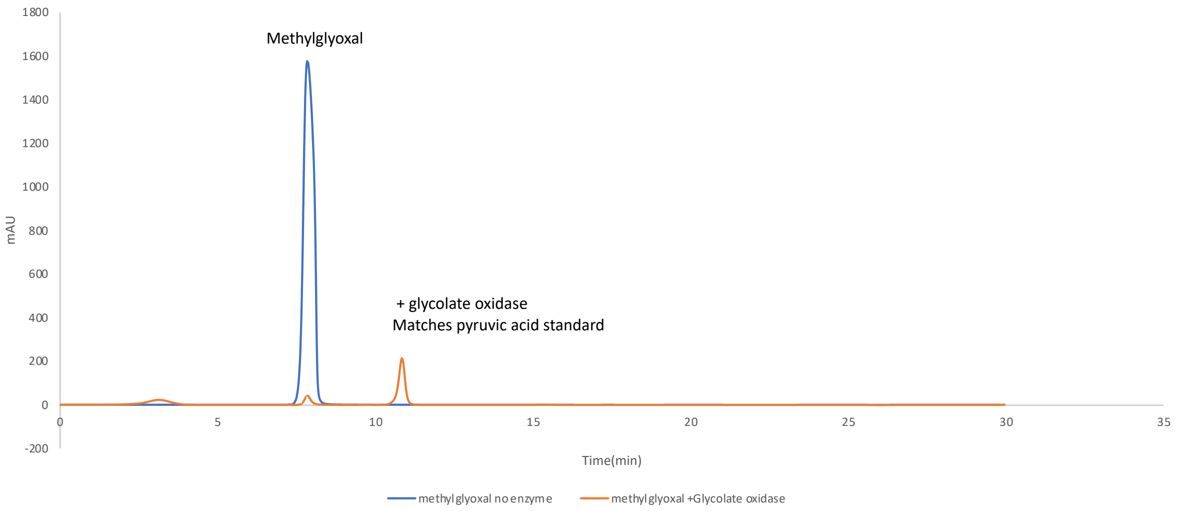

Figure S4C. HPLC analysis of reaction products from incubation of methylglyoxal with *Rhodococcus jostii* RHA1 glycolate oxidase enzyme. Aminex HPX-87H Organic Acids column (Bio-Rad) at 45°C, eluent 5 mM sulfuric acid flow rate 0.5 mL/min.

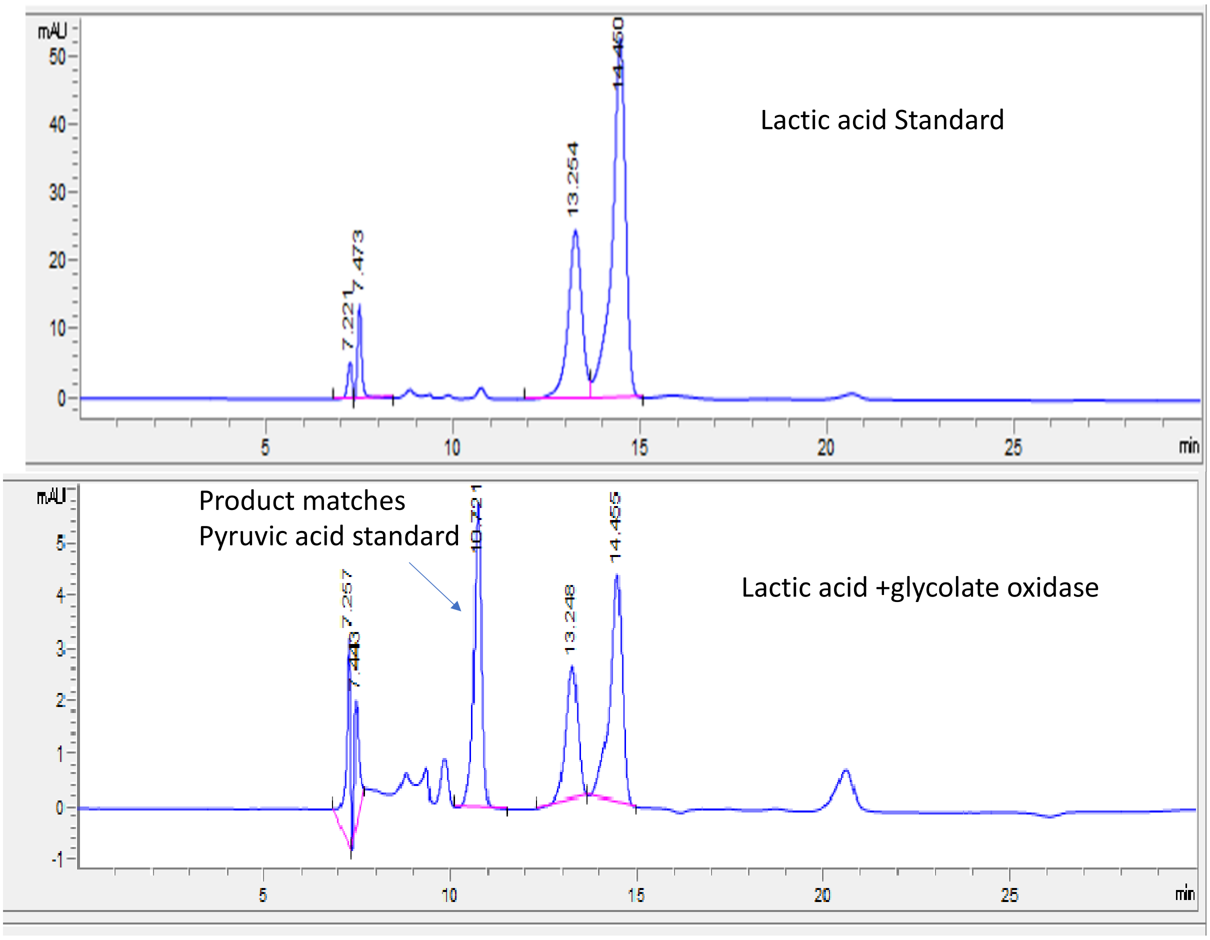

Figure S4D. HPLC analysis of reaction products from incubation of lactic acid with *Rhodococcus jostii* RHA1 glycolate oxidase enzyme. Aminex HPX-87H Organic Acids column (Bio-Rad) at 45°C, eluent 5 mM sulfuric acid flow rate 0.5 mL/min.

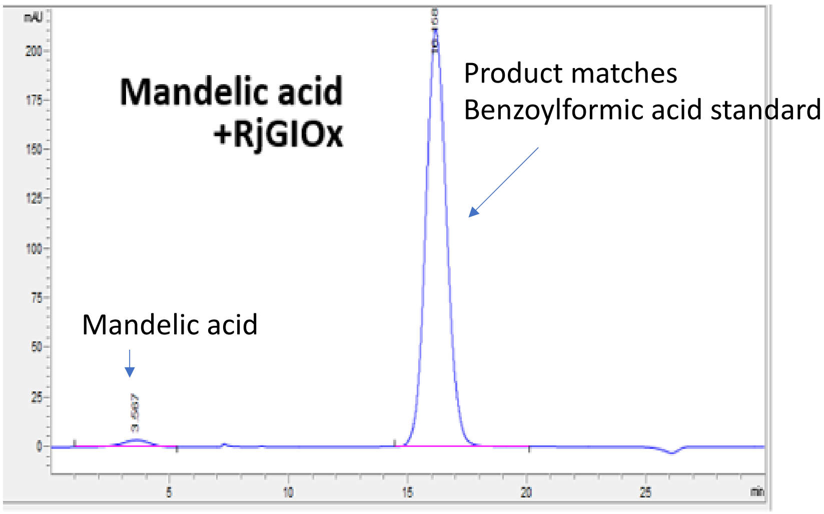

Figure S4E. HPLC analysis of reaction products from incubation of mandelic acid with *Rhodococcus jostii* RHA1 glycolate oxidase enzyme. Aminex HPX-87H Organic Acids column (Bio-Rad) at 45°C, eluent 5 mM sulfuric acid flow rate 0.5 mL/min.

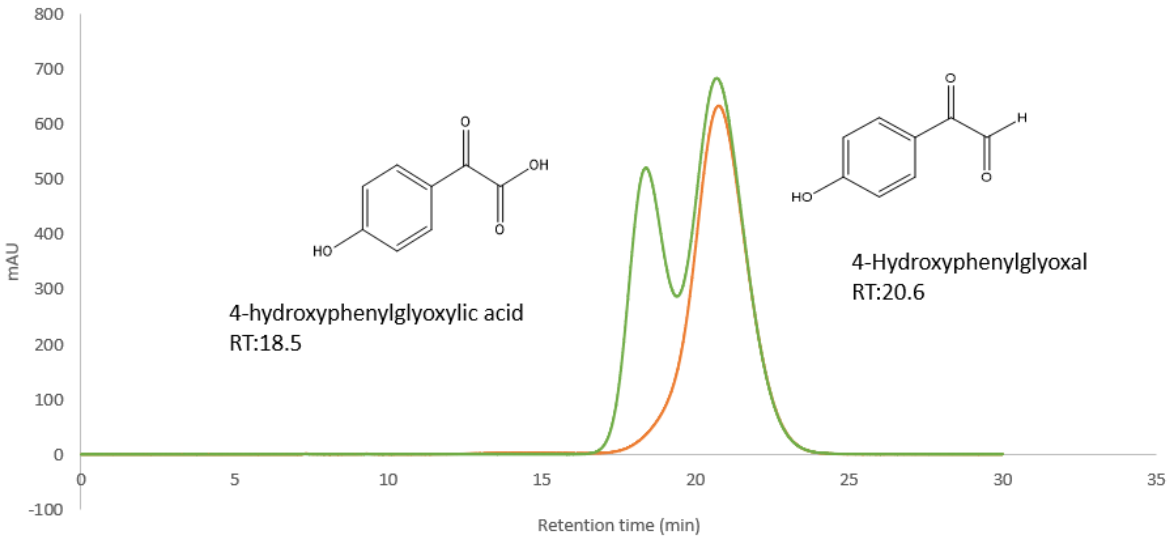

Figure S4F. HPLC analysis of reaction products from incubation of 4-hydroxyphenylglyoxal with *Rhodococcus jostii* RHA1 glycolate oxidase enzyme. Green line, sample treated with *R. jostii* RHA1 glycolate oxidase; orange line, control lacking glycolate oxidase enzyme. Reverse phase column Hyperclone 5u BDS C18 column, flow rate 0.5 mL/min, monitoring at 270 nm. Gradient: water (A) and methanol (B) containing 1% formic acid; 5% B (0–5 min); 5–10% B (5–10 min); 10–75% B (10–35 min); 75–100% B (35– 40 min); 100% MeOH (40–45 min); 100 to 10% B (45–50 min).

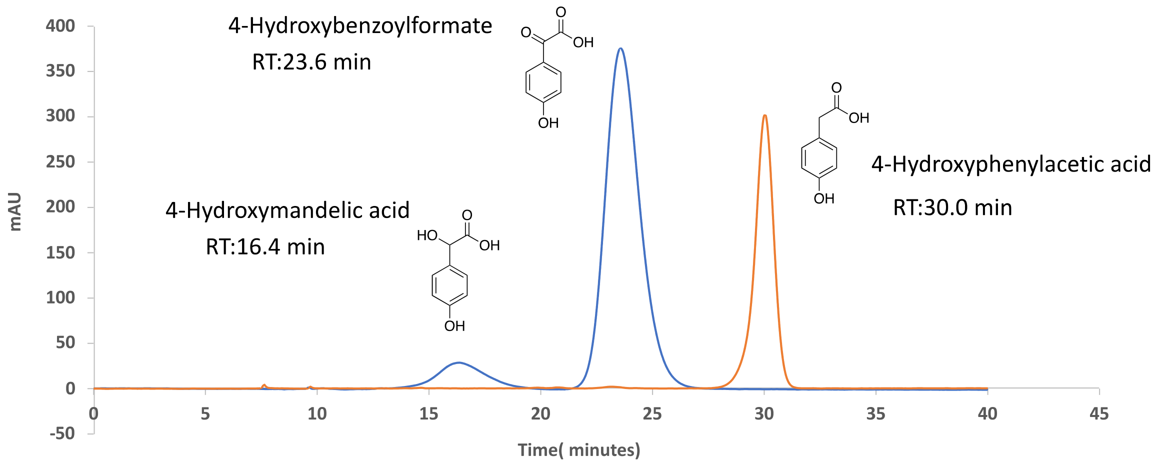

Figure S4G: HPLC analysis of reaction products from incubation of 4-hydroxyphenylacetic acid with *Rhodococcus jostii* RHA1 glycolate oxidase enzyme. Blue line, sample treated with *R. jostii* RHA1 glycolate oxidase; orange line, control lacking glycolate oxidase enzyme. Reaction mixtures were separated on an Aminex HPX-87H Organic Acids column (300 x 7.8 mm) (Bio-Rad) at 45°C, with 5 mM sulfuric acid as mobile phase and a flow rate of 0.5 mL/min.

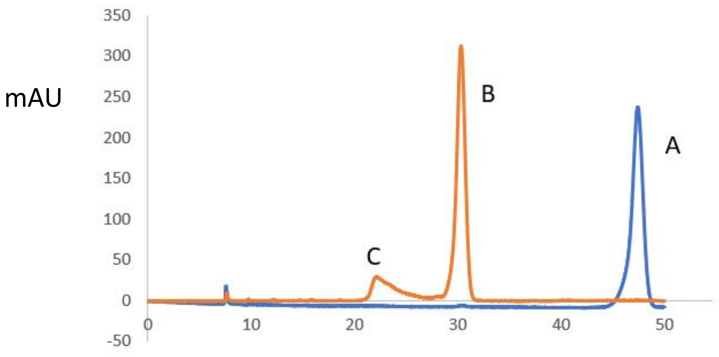

Figure S5. Conversion of 2mM HMF to FDCA by 90 µg *R. jostii* RHA1 glycolate oxidase and 40 mg catalase in 1 ml scale after 24hr at 30 ^{o}C (blue line, control HMF; orange line, after incubation with RjGlOx and catalase), showing complete conversion of HMF (peak A) to HMFCA (peak B) and 2,5-FDCA (peak C), with HMFCA as the major product.

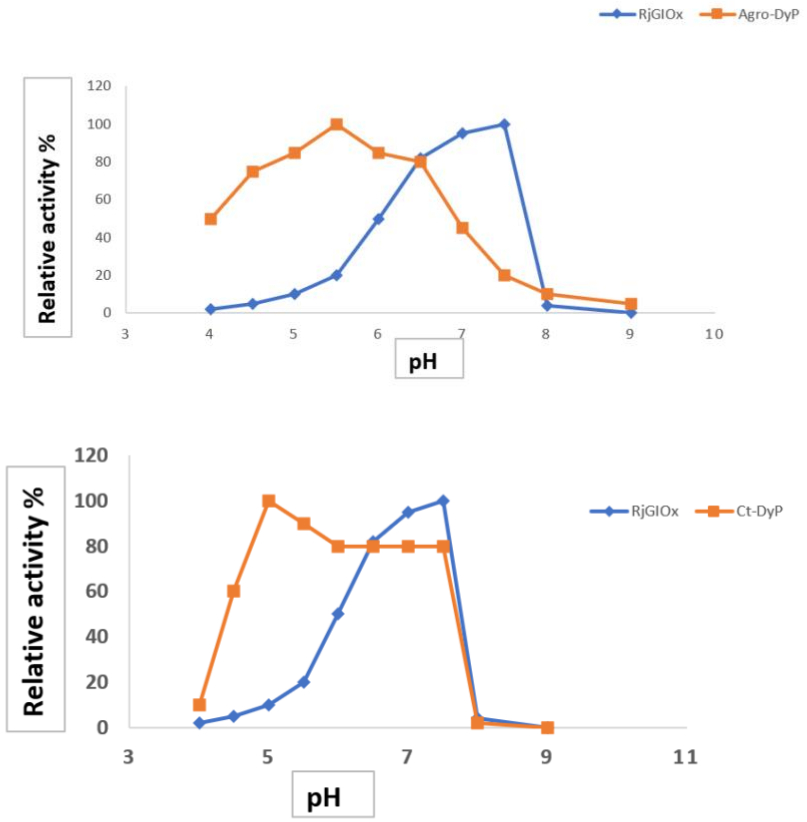

Figure S6. Activity vs pH for *Agrobacterium* DyP and *Comamonas testosteroni* DyP compared with *R. jostii* glycolate oxidase

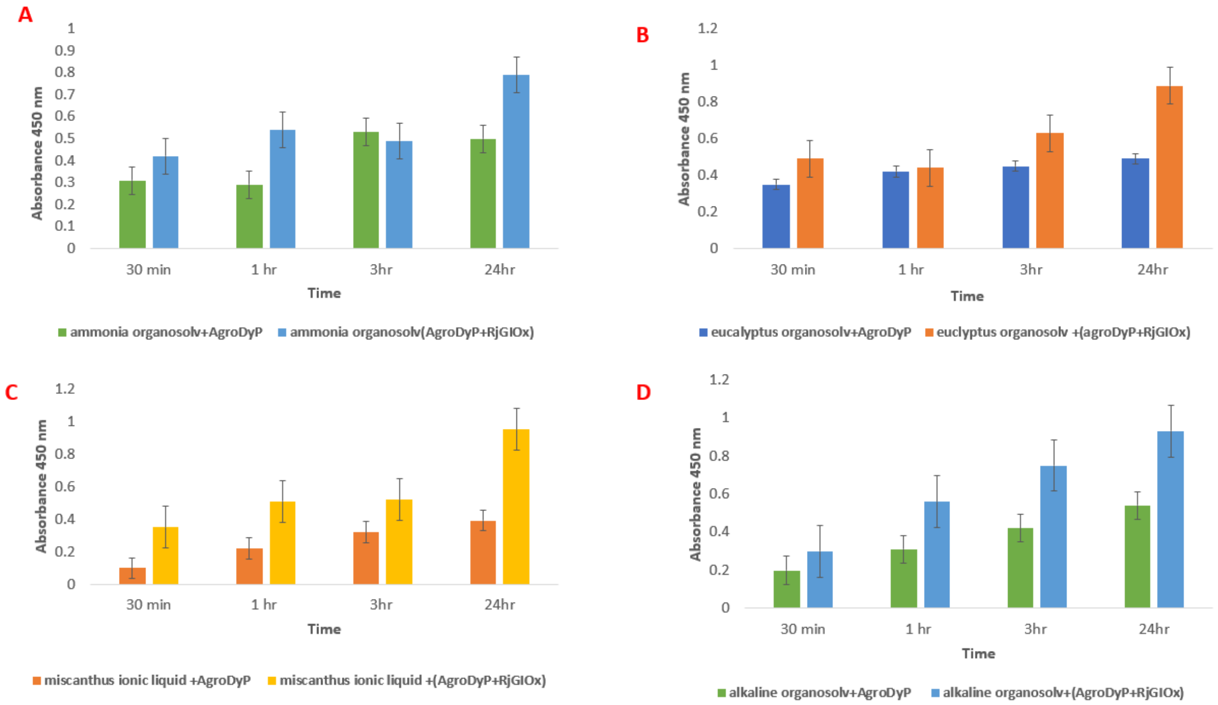

Figure S7. Assays of low molecular weight ketone/aldehyde formation (DNP assay) from conversion of polymeric lignins by AgroDyP/1 mM H_2O_2 vs AgroDyP + RjGlOx/1 mM glycolic acid. A, ammonia organosolv lignin; B, eucalyptus organosolv lignin; C, miscanthus ionic liquid lignin; D, alkaline organosolv lignin.

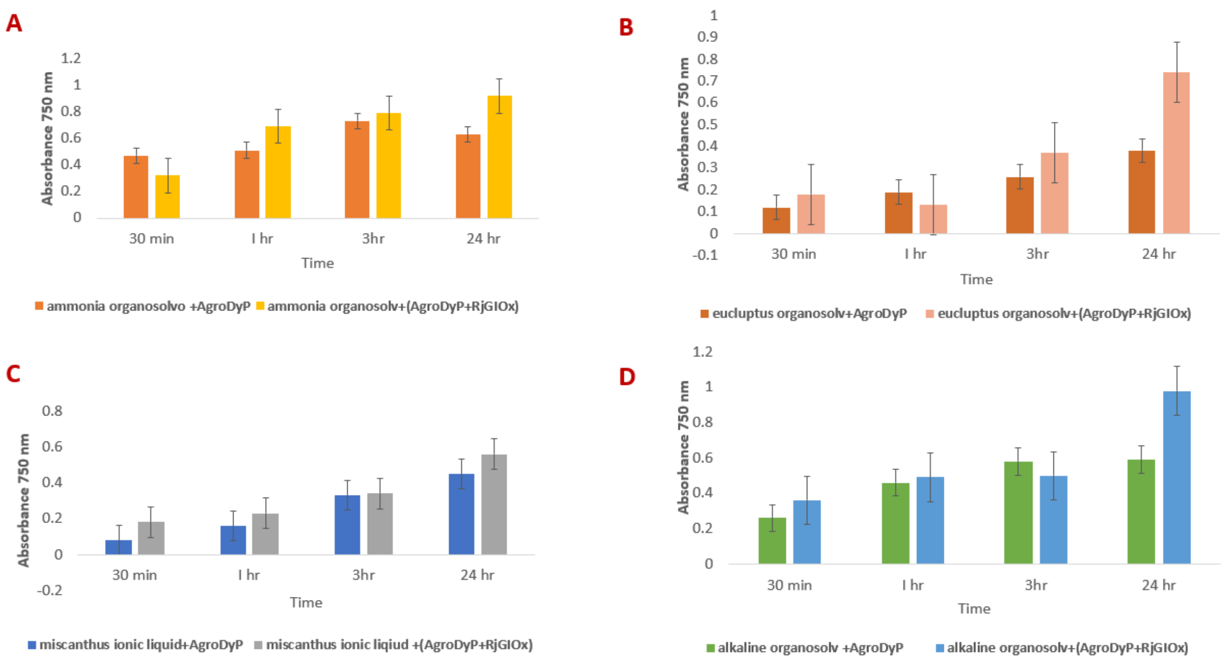

Figure S8. Assays of low molecular weight phenolic product formation (FCA assay) from polymeric lignins by AgroDyP/1 mM H_2O_2 vs AgroDyP + RjGlOx/1 mM glycolic acid. A, ammonia organosolv lignin; B, eucalyptus organosolv lignin; C, miscanthus ionic liquid lignin; D, alkaline organosolv lignin.

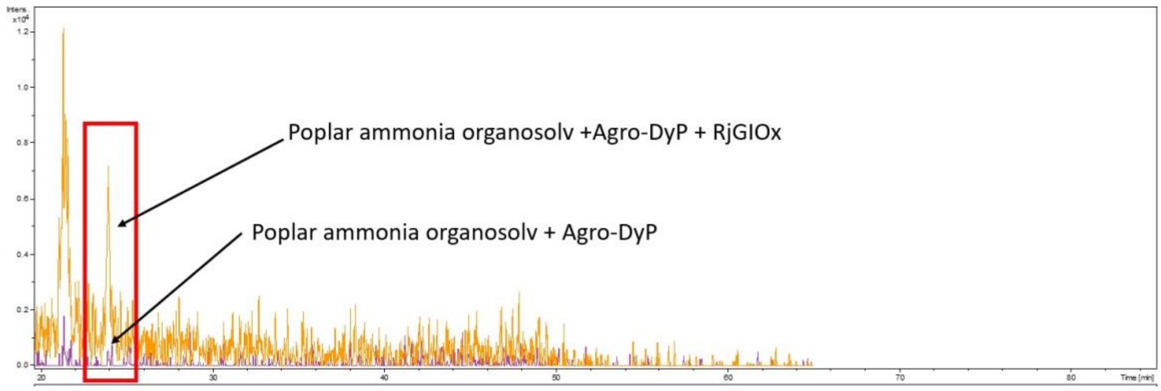

Figure S9A: Extracted ion chromatogram at *m/z* 169 for peak P2 at retention time 24.5 min, identified as vanillic acid (MH$^+$ 169).

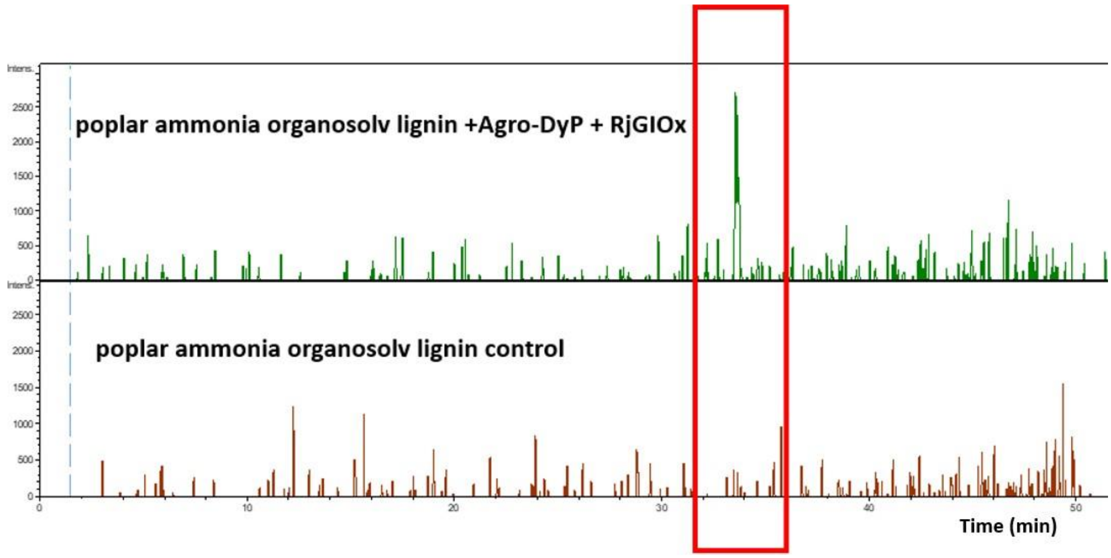

Figure S9B. Extracted ion chromatogram at *m/z* 243 for peak P3 at retention time 33.1 min. Proposed structure 1-(4-hydroxy-3,5-dimethoxyphenyl)-2,3-dihydroxypropan-1-one (MNa$^+$ 243).

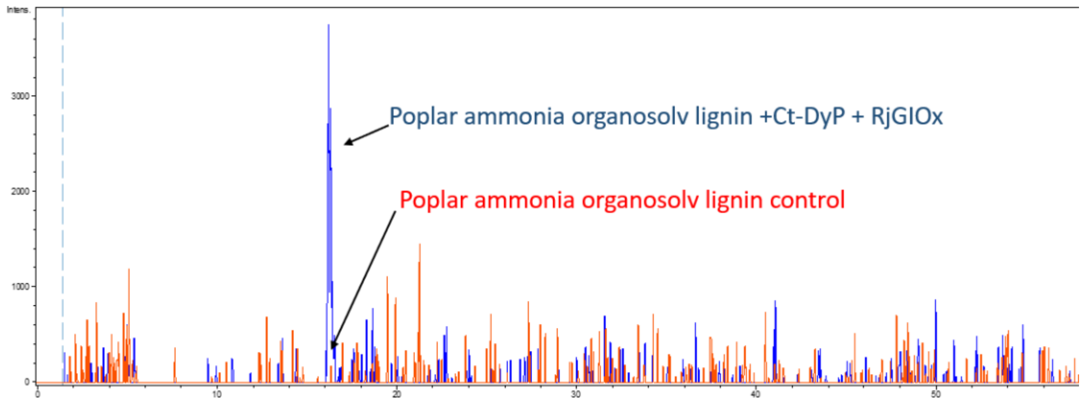

Figure S9C. Extracted ion chromatogram at *m/z* 125 for peak P6 at retention time 16 min, identified as guaiacol (MH$^+$ 125).

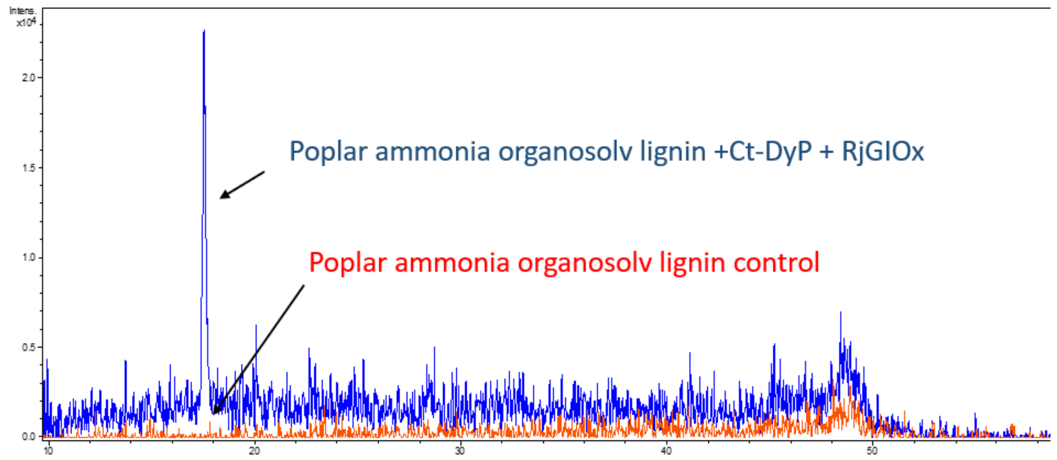

Figure S9D: Extracted ion chromatogram at *m/z* 153 for peak P7 at retention time 17.2 min, identified as 4-hydroxy-3-methoxy benzaldehyde (vanillin, MH^+ 153).

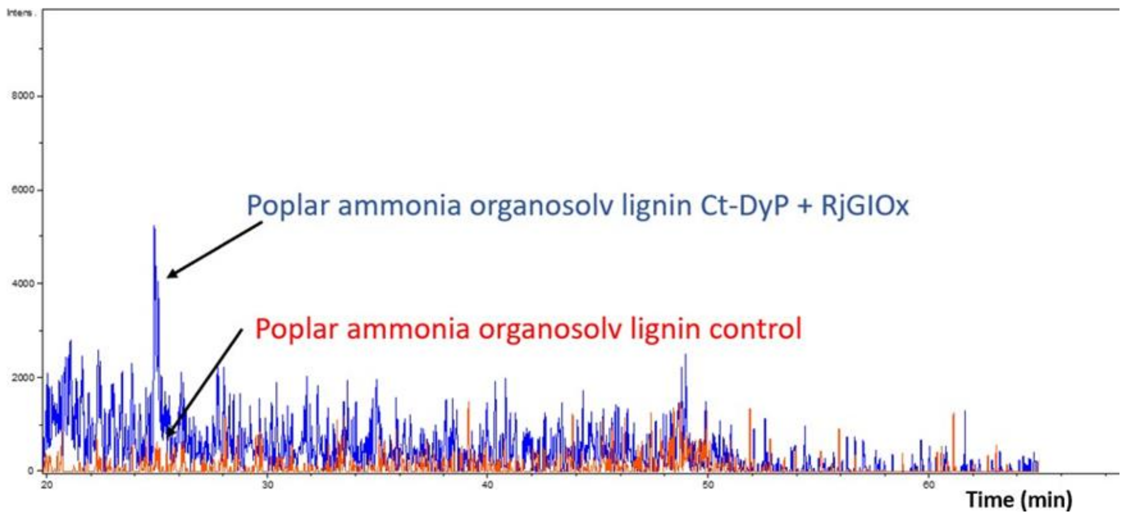

Figure S9E: Extracted ion chromatogram at *m/z* 155 for peak P8 at retention time 24.4, identified as protocatechuic acid (MH^+ 155).

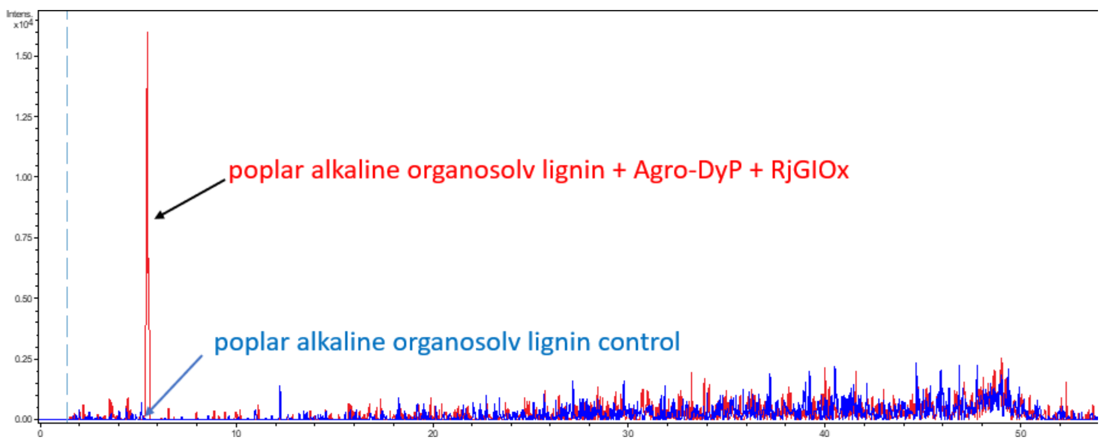

Figure S9F: The extracted ion chromatogram at *m/z* 99 for peak P10 at retention time 5.5 min, unidentified compound.

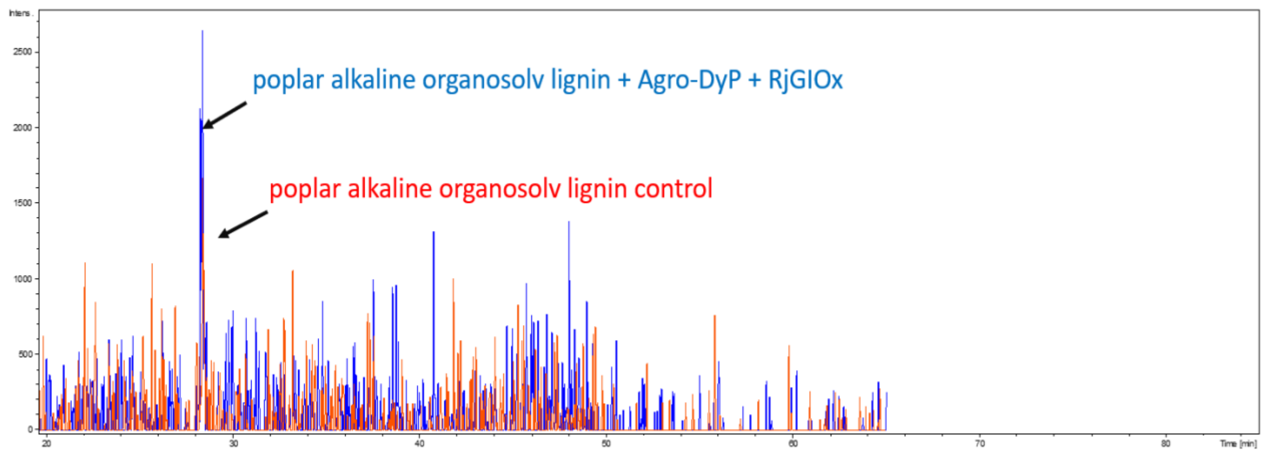

Figure S9G: The extracted ion chromatogram at *m/z* 146 for peak P14 at retention time 29 min, unidentified compound.

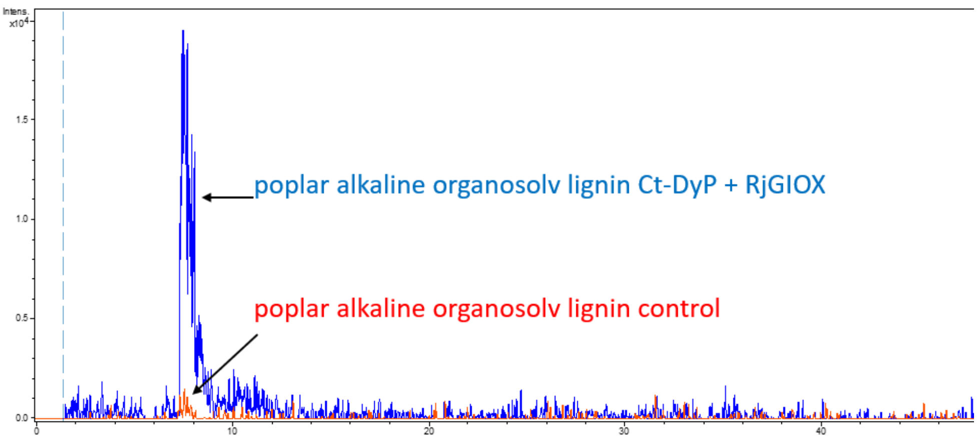

Figure S9H: The extracted ion chromatogram at *m/z* 157 for peak P16 at retention time 8.7min , proposed structure 2-methoxy-6-hydroxy-1,4-hydroquinone (MH^+ 157).

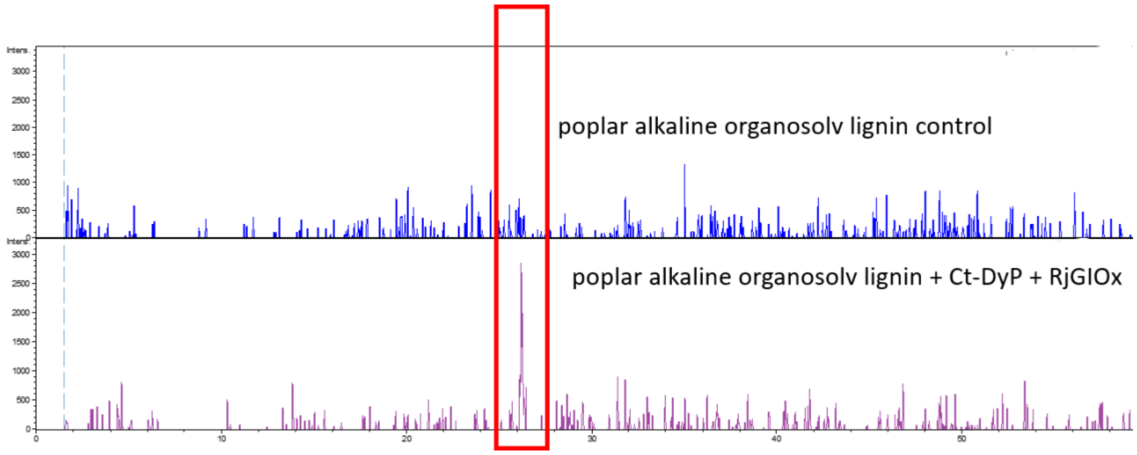

Figure S9I. Extracted ion chromatogram at *m/z* 183 for peak P20 at retention time 26.3 min, identified as 4-hydroxy-3,5-dimethoxybenzaldehyde (syringaldehyde).

A. Products from organosolv lignin	Concentration in reaction mixture
Glyoxylic acid	0.029 mM
Guaiacol	0.008 mM
Vanillin	0.02 mM
Protocatechuic acid	0.04 mM
Vanillic acid	0.11

B. Products from Avantium humins	Concentration in reaction mixture
HMF	2.6 mM
FDCA	0.09 mM
FFCA	0.12 mM

Figure S10 Estimated concentrations of reaction product peaks in bioconversion of (A) poplar alkaline organosolv lignin (B) Avantium humins by AgroDyP and *R. jostii* glycolate oxidase, by comparison with authentic standards via LC-MS.

Figure S11. Authentic standards used for identification of reaction products.

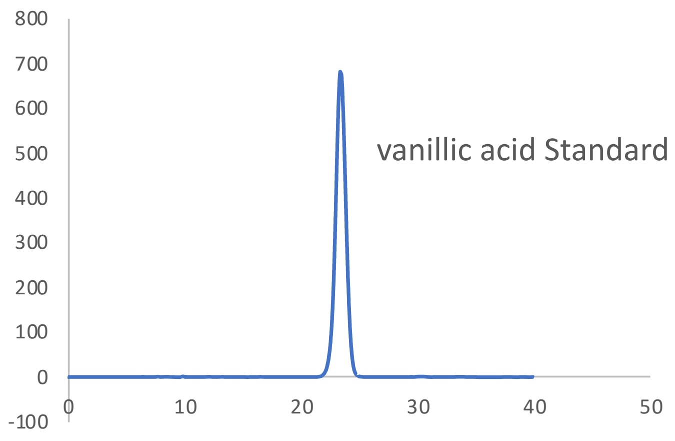

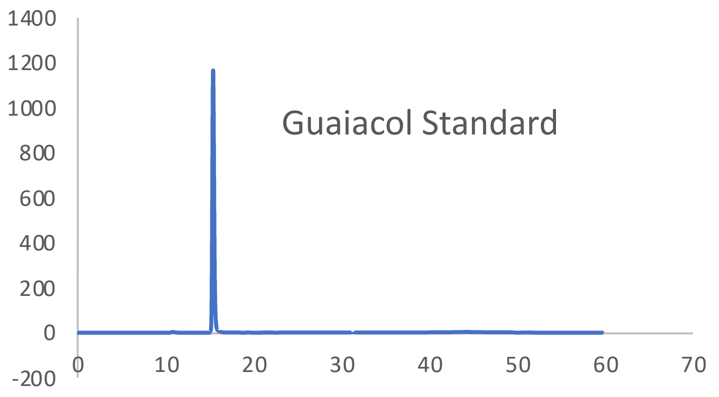

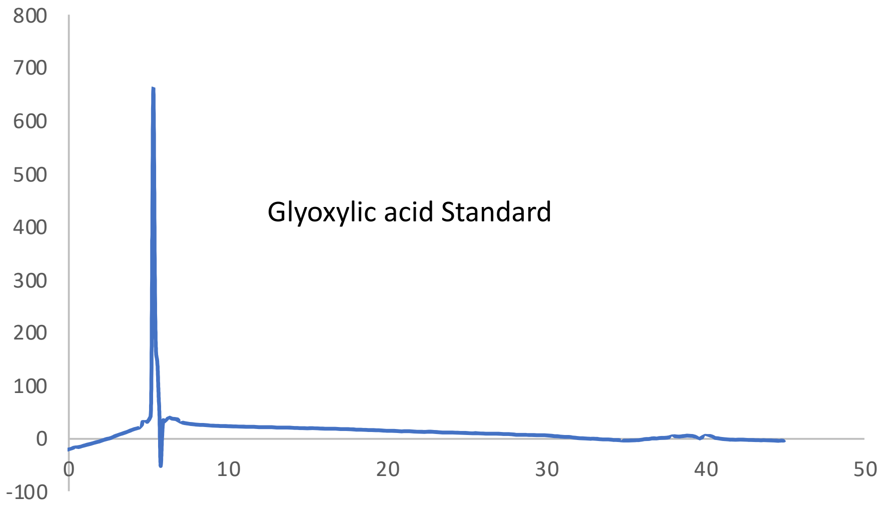

800
700
600
500
400
300
200
100
0
-100
0
10
20
30
40
50
Glyoxylic acid Standard

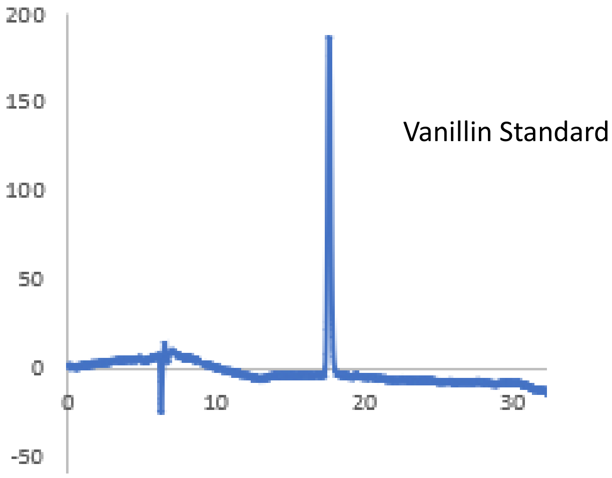

200
150
100
50
0
-50
0
10
20
30
Vanillin Standard

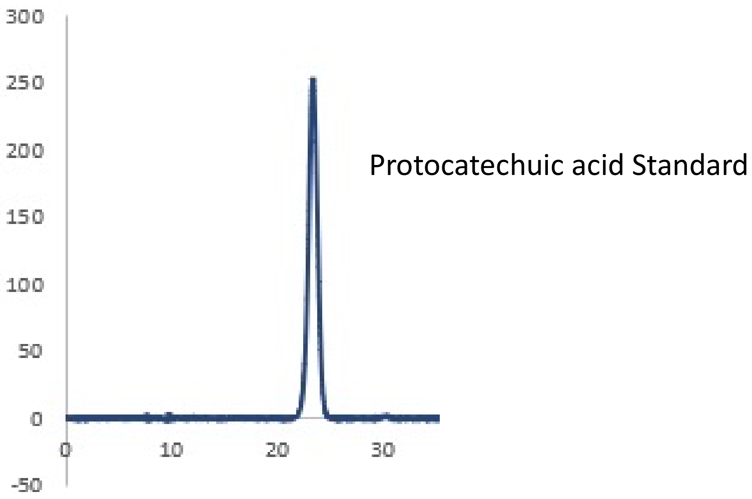

300
250
200
150
100
50
0
-50
0
10
20
30
Protocatechuic acid Standard

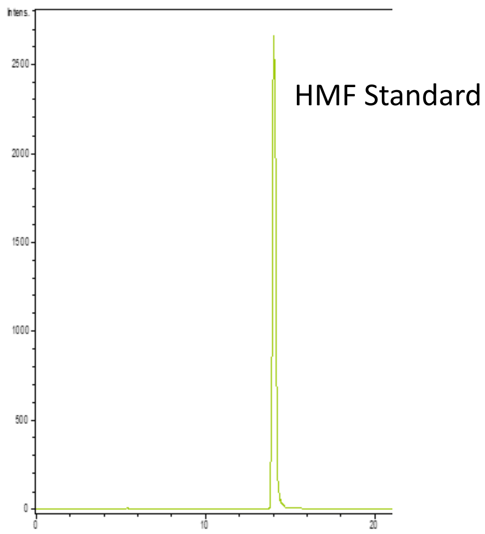
Intens.
2500
2000
1500
1000
500
0
0
10
20
HMF Standard

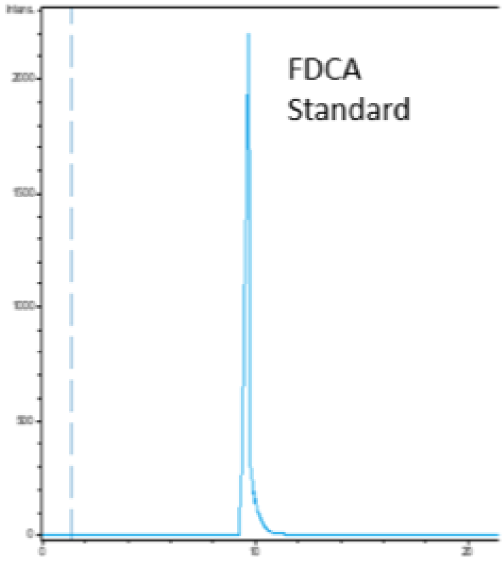
FDCA
Standard

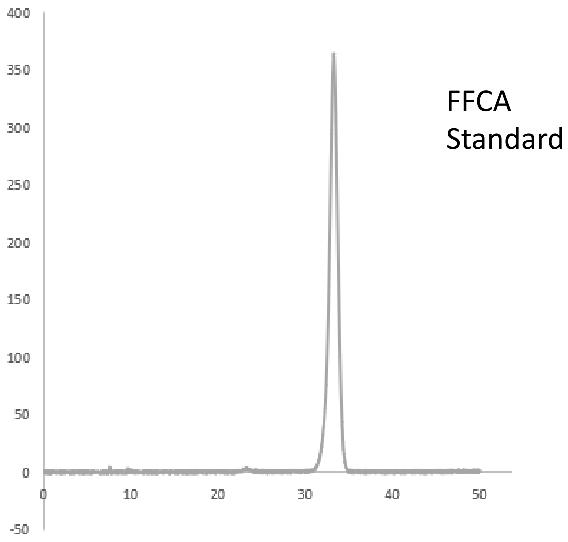
400
350
300
250
200
150
100
50
0
-50
0
10
20
30
40
50
FFCA
Standard

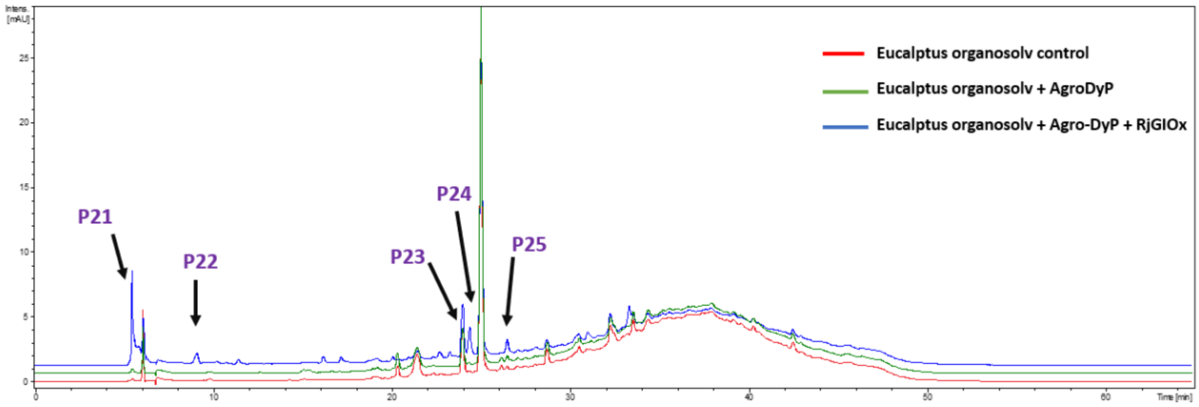

Figure S12A: Total ion chromatogram for bioconversion of eucalyptus organosolv lignin by Agro-DyP and RjGlOx. (red line, lignin control; green line, lignin and Agro-DyP; blue line, lignin, Agro-DyP and RjGlOx). New or enhanced peaks are marked. P21 5.5 min *m/z* 99, identical to P1,P10; P22 9.2 min *m/z* 210, unknown; P23 24.3 min *m/z* 155 protocatechuic acid; P24 24.5 min *m/z* 169 vanillic acid; P25 26.5 min unknown.

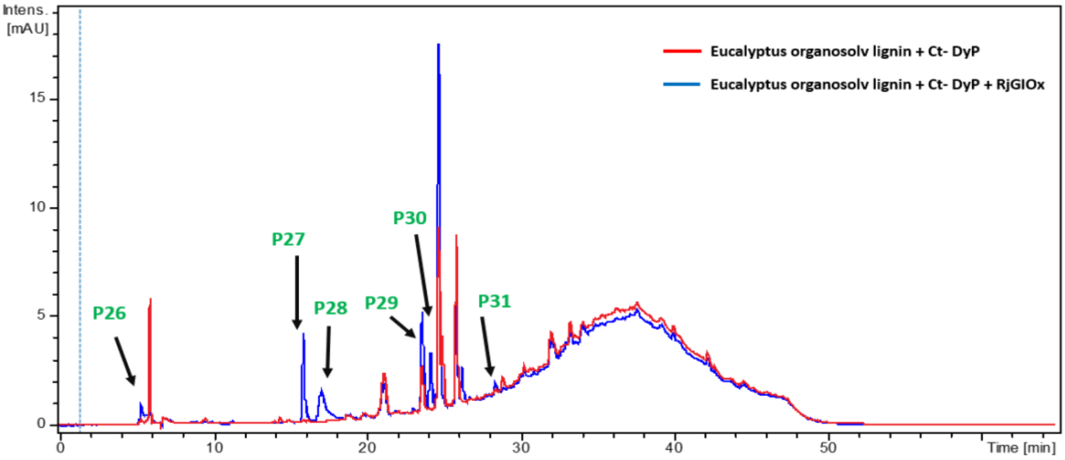

Figure S12B: Total ion chromatogram for bioconversion of eucalyptus organosolv lignin by Ct-DyP and RjGlOx (red line, lignin with Ct-DyP; blue line, Ct-DyP and RjGIOx). New or enhanced peaks are marked. P26 5.5 min *m/z* 99, identical to P1,P10; P27 16.0 min *m/z* 125, guaiacol; P28 17.4 min *m/z* 153 vanillin; P29 24.3 min *m/z* 155 protocatechuic acid; P30 24.5 min *m/z* 169 vanillic acid; P31 28.0 min *m/z* 199 identified as syringic acid (see below).

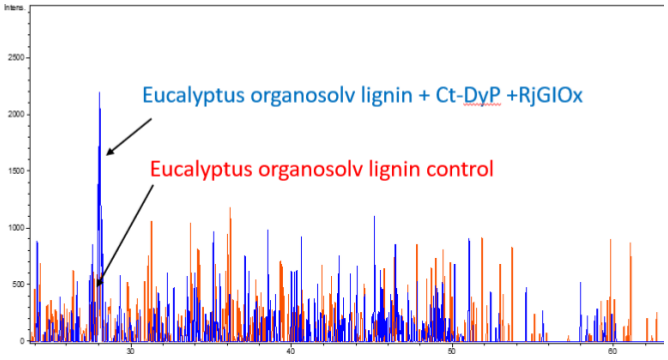

Figure S12C: Extracted ion chromatogram at *m/z* 199 for peak P31 at retention time 28.2 min, identified as 4-hydroxy-3,5-dimethoxybenzoic acid (MH^+).

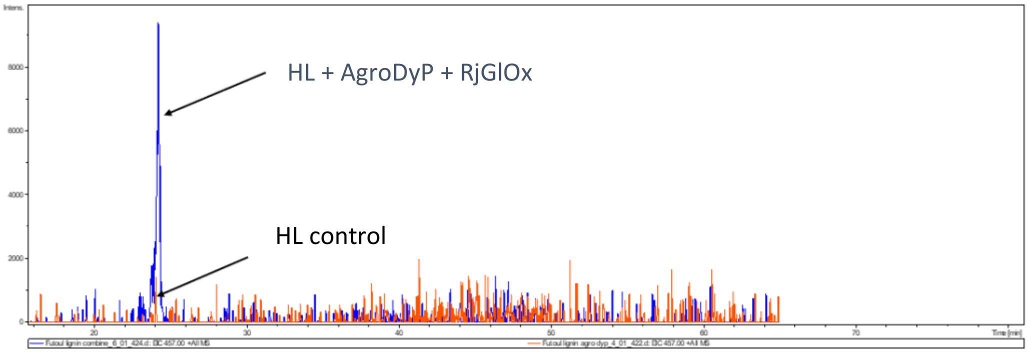

Figure S13A: Extracted ion chromatogram at *m/z* 227 for peak F2 from incubation of HL (red line) with Agro-Dyp enzyme in the presence of RjGlOx (blue line). Identified as 3- hydroxy-1-(4-hydroxy-3,5-dimethoxyphenyl) propan-1-one (MH^+).

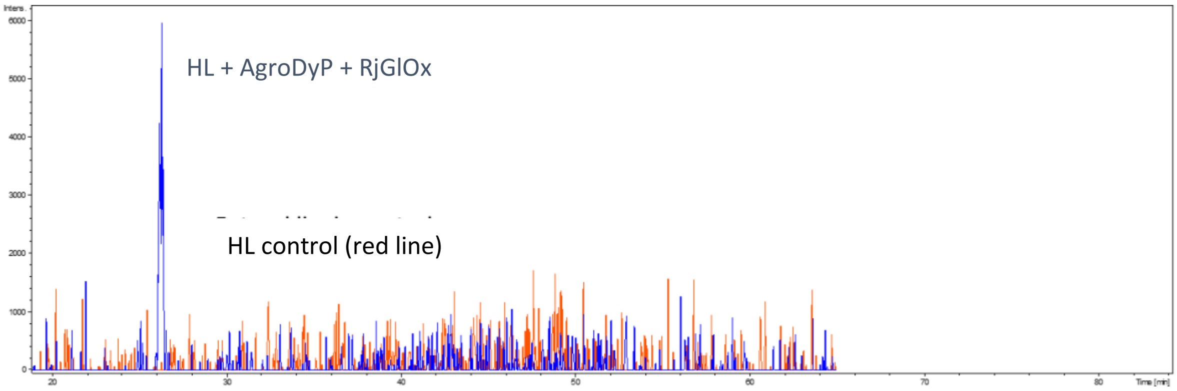

Figure S13B: Extracted ion chromatogram at *m/z* 183 for peak F3 from incubation of HL (red line) with Agro-Dyp enzyme in the presence of RjGlOx (blue line), identified as 4-hydroxy-3,5-dimethoxybenzaldehyde (MH^+).

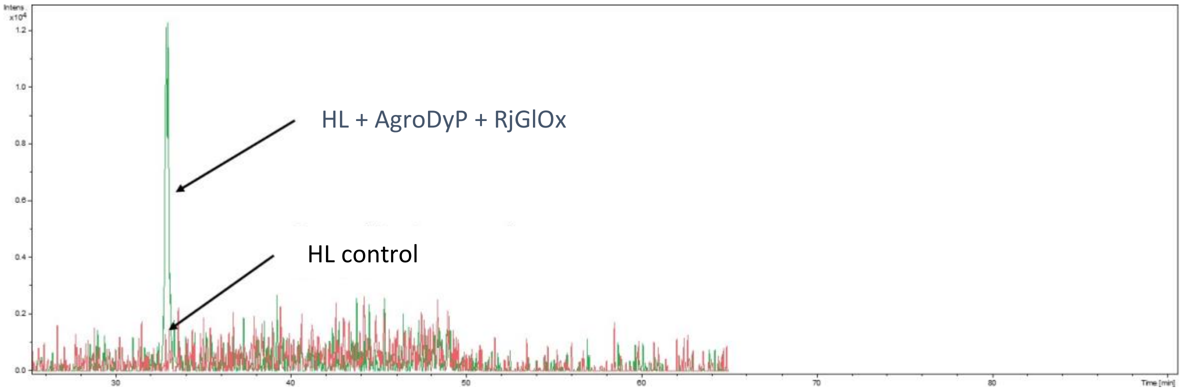

Figure S13C: Extracted ion chromatogram at m/z 243 for peak F4 from incubation of HL (red line) with Agro-Dyp enzyme in the presence of RjGlOx (green line), identified as 2,3-dihydroxy-1-(4-hydroxy-3,5-dimethoxyphenyl) propan-1-one (MH^+).

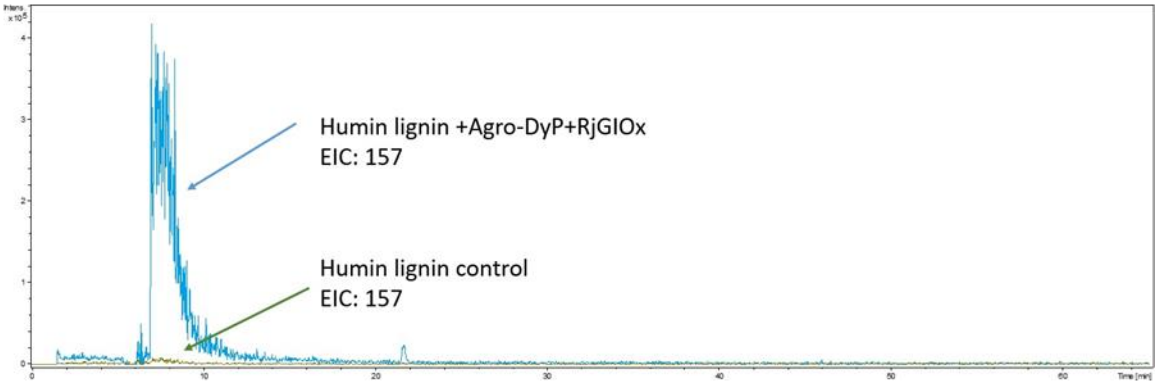

Figure S14A: Extracted ion chromatogram at *m/z* 157 for peak H1 from incubation of humins (green line) with Agro-Dyp enzyme in the presence of RjGlOx (blue line). Identified as 2,5-furandicarboxylic acid (MH^+) compared to authentic compound.

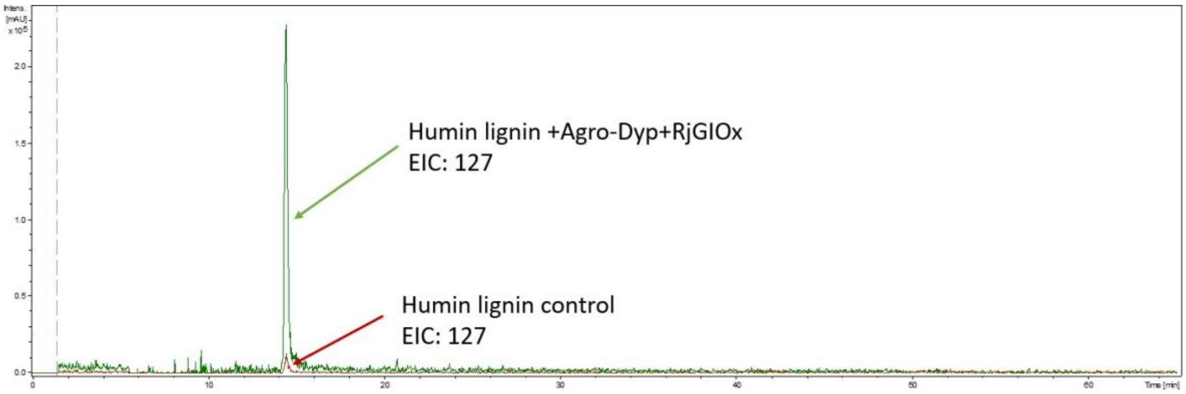

Figure S14B: Extracted ion chromatogram at *m/z* 127 from peak H2 from incubation of humins (red line) with Agro-Dyp enzyme in the presence of RjGlOx (green line), identified as 5-hydroxymethylfurfural (MH^+) compared to authentic compound.

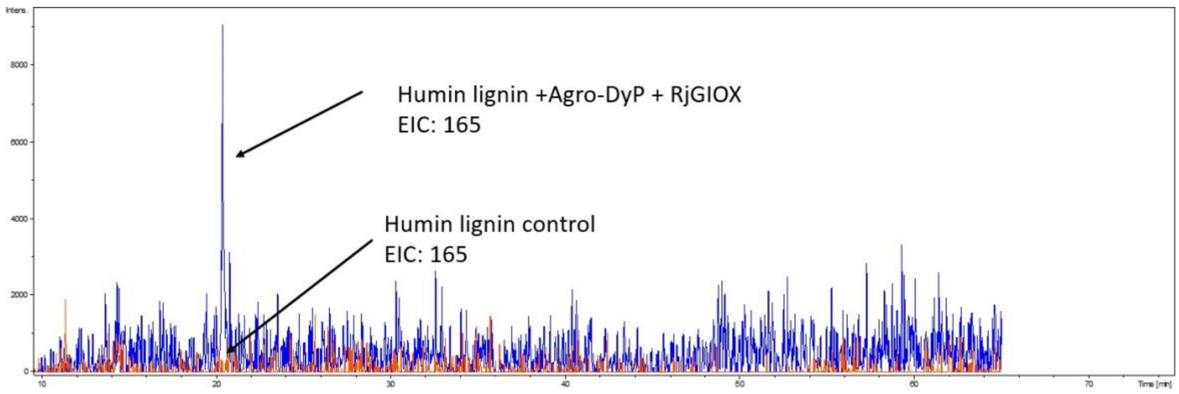

Figure S14C: Extracted ion chromatogram at *m/z* 165 for peak H3 from incubation of humins (red line) with Agro-Dyp enzyme in the presence of RjGlOx (blue line). Identified as 5-hydroxymethyl-2- furancarboxylic acid (MNa^+) compared to authentic compound.

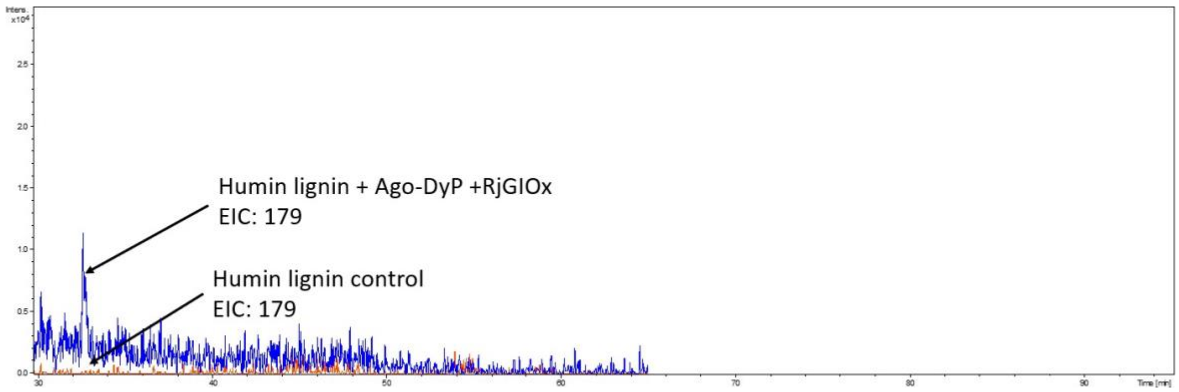

Figure S14D : Extracted ion chromatogram for *m/z* 179 for peak H7 from incubation of humins (red line) with Agro-Dyp enzyme in the presence of RjGIOx (blue line), identified as 5-formyl-2- furancarboxylic acid (MK^+) at retention time 33min compared to authentic compound.
